# Evaluating Variant Calling Tools for Non-Matched Next-Generation Sequencing Data

**DOI:** 10.1038/srep43169

**Published:** 2017-02-24

**Authors:** Sarah Sandmann, Aniek O. de Graaf, Mohsen Karimi, Bert A. van der Reijden, Eva Hellström-Lindberg, Joop H. Jansen, Martin Dugas

**Affiliations:** 1 Institute of Medical Informatics, University of Münster, Münster, 48149, Germany; 2 Laboratory Hematology, RadboudUMC, Nijmegen, 6525, Netherlands; 3 Center for Hematology and Regenerative Medicine, Department of Medicine Huddinge, Karolinska Institutet, Stockholm, 14186, Sweden

## Abstract

Valid variant calling results are crucial for the use of next-generation sequencing in clinical routine. However, there are numerous variant calling tools that usually differ in algorithms, filtering strategies, recommendations and thus, also in the output. We evaluated eight open-source tools regarding their ability to call single nucleotide variants and short indels with allelic frequencies as low as 1% in non-matched next-generation sequencing data: GATK HaplotypeCaller, Platypus, VarScan, LoFreq, FreeBayes, SNVer, SAMtools and VarDict. We analysed two real datasets from patients with myelodysplastic syndrome, covering 54 Illumina HiSeq samples and 111 Illumina NextSeq samples. Mutations were validated by re-sequencing on the same platform, on a different platform and expert based review. In addition we considered two simulated datasets with varying coverage and error profiles, covering 50 samples each. In all cases an identical target region consisting of 19 genes (42,322 bp) was analysed. Altogether, no tool succeeded in calling all mutations. High sensitivity was always accompanied by low precision. Influence of varying coverages- and background noise on variant calling was generally low. Taking everything into account, VarDict performed best. However, our results indicate that there is a need to improve reproducibility of the results in the context of multithreading.

Recent developments in next-generation sequencing (NGS) platforms has revolutionized the application of personalized medicine. Due to improvements with respect to time and costs^1^,^2^ compared to Sanger sequencing^3^, targeted sequencing can now be performed as part of clinical routine^4^. In addition, NGS provides a technique that is able to call variants with allelic frequencies below 20%, which is the detection limit of Sanger sequencing^5^.

NGS is helping physicians and researches to understand the evolution and progression of genetically related diseases, including cancer. In the past decades, numerous cancer driver genes and -pathways have been identified^6^. Subtypes of cancer could be defined^7^ and the prognostic relevance of several mutations could be established^8^ with the help of NGS.

However, for the application of NGS in clinical routine, it is essential to generate valid results. Both the presence and absence of mutations can influence a patient’s diagnosis, prognosis and therapy. Therefore, both high sensitivity and high positive predictive value (PPV) are required.

Sequencing errors leading to artefactual data are a common problem with basically all NGS platforms^9^,^10^,^11^,^12^. If there are no ultra-deep sequencing data available, it is often challenging to distinguish low-frequency mutations from random sequencing errors. Furthermore, the detection of variants in homopolymeric or other repetitive regions can be distinctly challenging^2^,^9^,^11^,^12^,^13^.

There are various tools for variant calling and they all aim to call variants in NGS data with high sensitivity and precision. Altogether we found more than 40 open-source tools that have been developed in the past eight years. The algorithms these tools use for calling single nucleotide variants (SNVs) and short indels (up to 30 bp, but usually shorter) can differ considerably. GATK^14^, Platypus^15^, FreeBayes^16^ and SAMtools^17^ rely on bayesian approaches. VarScan^18^ on the contrary uses a heuristic/statistical method to identify variants. SNVer^19^ relies on a frequentist approach, while LoFreq uses a Poisson-binomial distribution. Some tools like GATK or VarDict^20^ perform local realignment to improve indel calling. Usually the tools provide a set of parameters characterizing the reported variants and recommendations for filtration.

When considering the different variant calling tools, they all show superiority with certain configurations, on a selected set of samples and in comparison to a selected set of other tools. However, the analysed datasets are often simulated or result from healthy subjects. Previous studies, evaluating variant calling tools, usually compared only a small number of tools, considered matched-samples and/or whole genome or whole exome sequencing data^21^,^22^,^23^. To our knowledge, there is no comprehensive evaluation of variant calling tools, which is based on real non-matched targeted sequencing data collected in clinical routine considering all available open-source tools.

Therefore, we performed variant calling with respect to SNVs and short indels on two sets of real Illumina targeted sequencing data. The first set comprises data of 54 patients with myelodysplastic syndrome (MDS), sequenced on Illumina HiSeq, using HaloPlex target enrichment kit. The second set comprises 111 patients with MDS, sequenced on Illumina NextSeq, using TruSight DNA Amplicon Sequencing Panel. In addition, two sets of simulated Illumina targeted sequencing data with varying coverage and error profiles are evaluated to identify the conditions under which a certain variant calling tool performs superior than others. These sets cover 50 samples each. Illumina HiSeq, as well as Illumina NextSeq data are simulated. Eight variant callers – GATK, Platypus, VarScan, LoFreq, FreeBayes, SNVer, SAMtools and VarDict – are compared based on their ability to call true mutations, with allelic frequencies down to 1%, to automatically identify artefacts, to call polymorphisms as quality control parameter, reproducibility of the results, output comparability and run time.

## Results

### Variant calling

#### Mutations and artefacts in the real datasets

Different from polymorphisms (for the analysis of polymorphisms see Supplementary Section S2.1), somatic mutations may be present in the samples with frequencies considerably lower than 0.50. Achieving high sensitivity as well as high PPV in this category is thus expected to be the actual challenge for the different variant calling tools compared. The number of mutations and artefacts reported by the eight tools in case of the first dataset (Illumina HiSeq, 54 samples) and of the second dataset (Illumina NextSeq, 111 samples) is summarized in Table 1.

The first dataset contains 113 true mutations (on average: 2.09 mutations/sample). FreeBayes is the only variant caller that succeeds in reporting all 113 mutations. LoFreq and VarDict achieve comparably good results by reporting 111 (*sens* = 0.98), resp. 109 (*sens* = 0.96) mutations. Only 58 out of 113 mutations (51%) are consistently reported by all eight variant calling tools (see Supplementary Fig. S2).

When considering precision it becomes obvious that FreeBayes’ high sensitivity goes along with a very poor positive predictive value (*PPV* = 0.01). VarScan and VarDict on the contrary achieve comparably good results, reporting the lowest number of false positive calls (VarScan: 7, *PPV* = 0.92; VarDict: 9, *PPV* = 0.92).

Using the *F*_1_ score (*F*_1_ = 2 · *sens* · *PPV*/(*sens* + *PPV*)) to evaluate the overall performance of the eight variant callers, considering both sensitivity and PPV with equal weight, VarDict performs best with respect to the first dataset (*F*_1_ = 0.94). Despite their relatively low sensitivity, VarScan (*F*_1_ = 0.83) and SNVer (*F*_1_ = 0.81) shows an overall good performance as well.

However, when regarding the second dataset generated on a different sequencing platform, considerable different observations are made. The dataset contains 226 mutations (on average: 2.04 mutations/sample). No tool succeeds in calling all 226 mutations – not even FreeBayes. VarDict achieves the highest sensitivity (*sens* = 0.91), followed by Platypus (*sens* = 0.83). Altogether only 21 out of 226 mutations (9%) are consistently reported by all eight tools (see Supplementary Fig. S3).

Due to the high number of false positive calls reported by every variant caller in the second dataset, the PPV is lower compared to the first dataset in all cases. VarScan (*PPV* = 0.46), GATK (*PPV* = 0.43) and SAMtools (*PPV* = 0.40) perform best regarding artefacts. The worst performance with respect to precision is observed using FreeBayes, as is the case in the first dataset (*PPV* = 0.01). Remarkably, SNVer (*PPV* = 0.03) and especially VarDict (*PPV* = 0.06), which showed exceptionally good precision when analysing the first dataset, are now among the worst tools.

The weaker performance with respect to sensitivity and precision leads to an overall decrease in the *F*_1_ score for all variant calling tools regarding the second dataset. Due to the highest number of false positive calls, FreeBayes performs worst according to the *F*_1_ score. Despite missing 70 true mutations, GATK shows the best results (*F*_1_ = 0.53).

**Figure 1:**
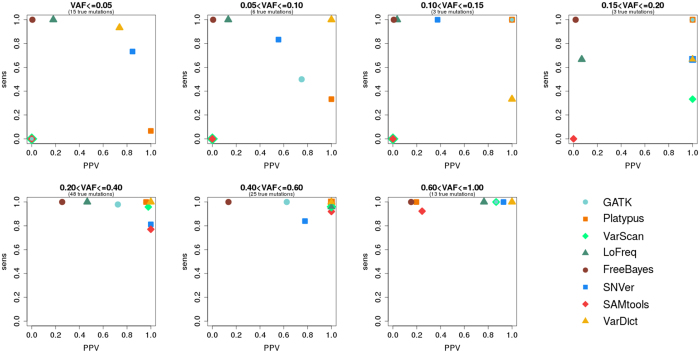
Sensitivity and PPV in relation to variant allele frequency (VAF) in case of GATK, Platypus, VarScan, LoFreq, FreeBayes, SNVer, SAMtools and VarDict analysing the first dataset (HiSeq).

Because we established that many mutations were missed and significant numbers of artefacts were reported by the different tools, we investigate sensitivity and PPV in relation to variant allele frequency. The results regarding the first dataset are displayed in Fig. 1.

It is obvious that LoFreq, FreeBayes, VarDict and SNVer are the only tools that are able to detect a majority of variants with allelic frequencies of 5% or below – although some exhibit a very low PPV at this level. GATK, VarScan and SAMtools do not succeed in calling any variants in this category. Platypus features *sens* ≤ 0.10.

We observe that GATK starts calling variants at 0.05 < *VAF* ≤ 0.10. VarScan starts calling variants at 0.15 < *VAF* ≤ 0.20. SAMtools, however, is not able to call any variants with allelic frequencies below 0.20 in the first dataset. Regarding the tools that already called variants at *VAF* ≤ 0.05, sensitivity is in most cases close to 1.00 if variants at higher allelic frequencies are considered.

Considering the rise of PPV with increasing VAF, huge differences between the eight variant calling tools compared can be observed. VarDict as well as SNVer feature *PPV* > 0.70 even if variants at *VAF* ≤ 0.05 are considered (Platypus is not considered due to its low sensitivity). While PPV of VarDict increases to 1.00 with higher VAFs, PPV of SNVer varies between 0.38 and 1.00. Except for the highest VAF category (0.60 < *VAF* ≤ 1.00), Platypus always features *PPV* > 0.95. GATK shows varying PPV between 0.62 and 1.00 if variants with allelic frequencies above 0.05 are considered. VarScan shows a continuously high PPV of at least 0.86 if variants of *VAF* > 0.15 are considered. A continuously low PPV can be observed in case of FreeBayes, with maximum PPV of 0.26 with 0.20 < *VAF* ≤ 0.40.

**Figure 2:**
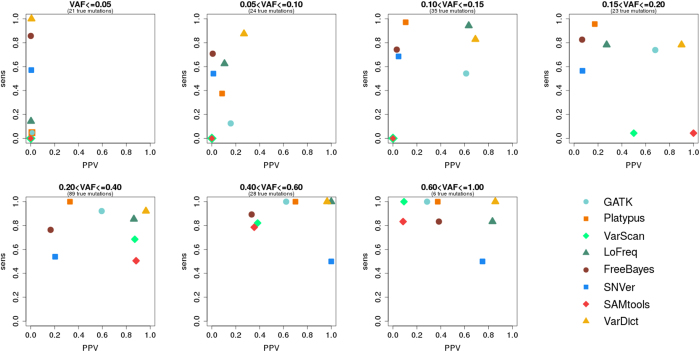
Relation between sensitivity, PPV and variant allele frequency (VAF) in case of GATK, Platypus, VarScan, LoFreq, FreeBayes, SNVer, SAMtools and VarDict analysing the second dataset (NextSeq).

We also investigate sensitivity and PPV in relation to variant allele frequency in the second dataset (see Fig. 2).

Again, FreeBayes and VarDict succeed in detecting a majority of variants with allelic frequencies of 5% or below, however again with very low PPV. SNVer and especially LoFreq show considerably lower sensitivity (SNVer: 0.57 compared to 0.73; LoFreq: 0.14 compared to 1.00). As was the case for the first dataset, GATK, VarScan and SAMtools do not succeed in calling any variants in this category. Platypus features *sens* ≤ 0.05.

Analysis of the rise of sensitivity in the context of increasing allele frequency generally underscores the results of the first dataset. However, different from the first dataset, SNVer shows a consistently worse performance with respect to sensitivity. Without having a clear tendency, values fluctuate between 0.50 and 0.68. LoFreq only performs bad on variants with *VAF* ≤ 0.05.

In general a rise in PPV can be observed when VAF increases. At *VAF* ≤ 0.05 all tools feature PPV close to zero. However, at 0.05 < *VAF* ≤ 0.10 *PPV* = 0.26 can already be observed in case of VarDict. If variants with allelic frequencies above 0.15 are considered, VarDict even features *PPV* > 0.85. Altogether, this appears to be the best performance regarding precision that we observed. SNVer and FreeBayes show a similar increase in PPV – although to lower values. With minor exceptions, a similar development can also be observed in case of GATK, Platypus and LoFreq.

#### Influence of varying coverages

**Figure 3:**
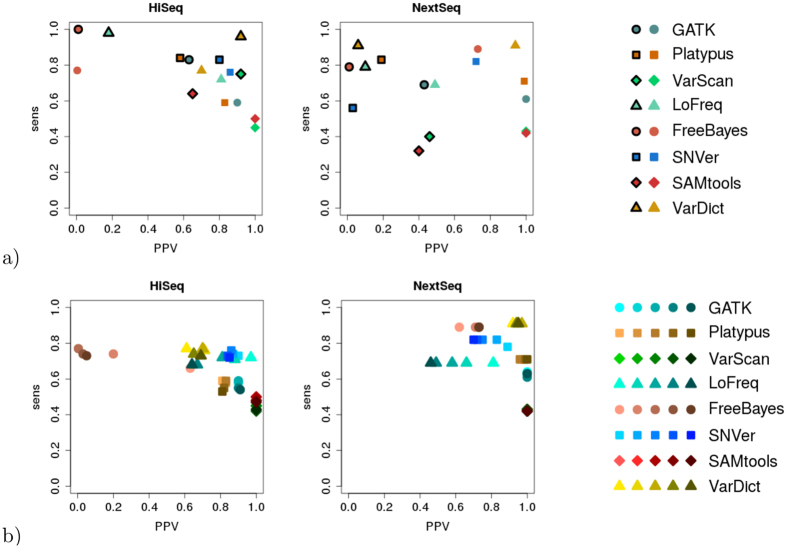
Relation between sensitivity and positive predictive value (PPV) in case of GATK, Platypus, VarScan, LoFreq, FreeBayes, SNVer, SAMtools and VarDict comparing (**a**) the real- and simulated datasets (real data: framed symbols) and (**b**) varying coverage in the simulated datasets (the darker the symbol, the higher the coverage).

To analyse the influence of varying coverages on sensitivity and PPV for the different variant calling tools, we simulate data with five different coverages: 500x, 1,000x, 1,983x (corresponds to first real dataset), 4,043x (corresponds to second real dataset) and 6,000x. However, before analysing the simulated data with varying coverages, we check the precision of our simulation by comparing sensitivity and PPV in the real- and corresponding simulated datasets (see Fig. 3a).

For both HiSeq and NextSeq data the sensitivity in the simulated datasets matches sensitivity in the real datasets well. With the simulated HiSeq data a general shift by ~0.20 to lower sensitivity can be observed. However, the relation between the different variant calling tools is almost identical. The only exceptions from this observation are SNVer, which performs slightly better than expected with simulated data, and VarScan, which performs slightly worse than expected with simulated data. Using the simulated NextSeq data, we see minor variations in sensitivity to both higher and lower values. A maximum difference is observed in case of SNVer (reality: *sens* = 0.56; simulation: *sens* = 0.82).

In contrast to sensitivity, real- and simulated datasets show considerable differences with respect to PPV. In general, a significantly higher PPV can be observed for simulated data in case of both datasets and all variant calling tools. The shift to higher PPVs is more distinct in case of the simulated NextSeq data. VarDict and FreeBayes are the only tools that show a decrease in PPV with simulated HiSeq data compared to real HiSeq data.

When regarding the varying coverages, we expect to observe a decrease in sensitivity as coverage decreases. Especially when considering mutations with low allelic frequencies, we expect calling to be more difficult in the context of low coverage.

The influence of varying coverages on the simulated datasets is shown in Fig. 3b (for the number of calls see Supplementary Tables S6 and S7). Contrary to our expectations, sensitivity of every caller appears to decrease slightly as coverage increases in case of simulated HiSeq data. In case of simulated NextSeq data, sensitivity of hardly any caller seems to be influenced by coverage. Platypus, LoFreq, FreeBayes and VarDict appear to be especially invariant towards changing coverage.

#### Influence of varying background noise

To analyse the influence of varying background noise on the performance of the eight variant calling tools, we determine the error profile of the original data and manipulate it to simulate data with three different levels of background noise: E1 (original error profile), E2 (doubled error rate), E3 (half error rate).

To investigate whether our simulation has been successful, we determine the level of background noise in the simulated datasets: HiSeq: *noise*_*E*3_ = 6.56 · 10^−3^, *noise*_*E*1_ = 1.81 · 10^−2^, *noise*_*E*2_ = 1.99 · 10^−2^; NextSeq: *noise*_*E*3_ = 9.89 · 10^−4^, *noise*_*E*1_ = 1.85 · 10^−3^, *noise*_*E*2_ = 3.57 · 10^−3^. Especially in case of simulated NextSeq data, the observed level of background noise matches our expectations: as simulated sequencing errors are doubled, doubling in the determined level of background noise is observed.

**Figure 4:**
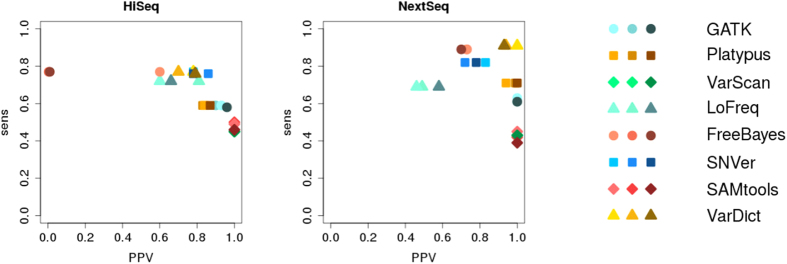
Relation between sensitivity and positive predictive value (PPV) in case of GATK, Platypus, VarScan, LoFreq, FreeBayes, SNVer, SAMtools and VarDict investigating varying levels of background noise in the simulated datasets (the darker the symbol, the higher the background noise).

The changes of sensitivity and PPV in the context of varying background noise in case of the two simulated datasets is shown in Fig. 4.

As background noise increases we expect to observe a minor decrease in sensitivity. An increased number of sequencing errors is expected to deteriorate alignment of the reads and, as a consequence, calling of low-frequency variants. Similarly, PPV is expected to decrease if background noise increases. Especially in case of highly sensitive variant callers like VarDict, LoFreq and FreeBayes, that are able to call variants with allelic frequencies below 0.05, an increased number of sequencing errors is expected to lead to an increased number of artefacts being called.

Figure 4 shows that the results of our simulation do not match our expectations in most cases. For simulated HiSeq data it is observed that FreeBayes is the only tool showing the expected increase in PPV as the level of background noise decreases. However, the increase can only be observed when comparing E3 with E2 and E1. An increase cannot be observed when comparing E2 and E1. All the other tools show no clear correlation of PPV and the level of background noise changes. With respect to sensitivity, all tools except for SAMtools appear to be invariant towards changes in the level of background noise. SAMtools does show the expected minor decrease in sensitivity as background noise increases.

Regarding simulated NextSeq data it can be observed that GATK and SAMtools are the only tools showing minor decrease in sensitivity as background noise increases. Figure 4 further indicates that with GATK, VarScan and SAMtools PPVs are invariant to changes in background noise. FreeBayes and VarDict show the expected minor deterioration in PPV as the level of background noise increases. Platypus and LoFreq however show a most unexpected increase in PPV with increasing background noise. No correlation between PPV and background noise can be identified in case of SNVer.

### Reproducibility

We investigate reproducibility of the variant calling pipelines by analysing the same dataset twice, with the same settings.

We indeed observe that all tools produce identical results. The only exception from this observation is VarDict. In case of the 54 samples sequenced on Illumina HiSeq, four out of 2,759 raw calls show minor differences with respect to the number of reference reads, alternate reads and the overall depth. One variant is called differently, which indicates that at least one of the calls is wrong (sample UPN1_15; first run: chr4:106,199,318 TAA>AATATAT; second run: chr4:106,199,320 A>AATATAT). Furthermore, one deletion is only reported in the first, but not in the second run (sample UPN1_30; chr12:12,046,729 delTATATATATATATATA). Regarding the 111 samples sequenced on Illumina NextSeq, five out of 12,196 calls show minor differences with respect to the number of reference reads, alternate reads and the overall depth.

Remarkably, all differences in the repeated variant calling with VarDict are observed in indels, but not SNVs. It is therefore likely that either the supervised or the unsupervised local realignment that is automatically performed by VarDict to improve the calling of indels contains a non-deterministic step.

We also investigate the influence of multithreading on the reproducibility of the results. GATK, Platypus and LoFreq are the only variant calling tools that provide options for multithreading. Comparing the results obtained from a single-thread run to those obtained from a multithreading run (eight threads), differences in the results are observed in case of all tools. The reported calls differ least when using Platypus. Some minor differences are detected only in case of the NextSeq dataset. Two calls differ in their “QUAL”-value and information provided in the “INFO” field. One call is not present in the first, but only in the second multithreading run.

In case of LoFreq, we observe differences with respect to the automatic filters “Minimum SNV Quality” and “Minimum Indel Quality”. Minimum values are not defined by our pipeline, but automatically calculated by the tool. The values differ for all samples. Furthermore, both filters are reported twice in case of the multithreading run – with different thresholds. In a majority of cases the thresholds reported in the multithreading run differ from those reported in the single-thread run. On average, filtration is stricter in case of the multithreading run. In the first dataset, 185 variants (5%) are only present in the first run using only one thread and 10 variants are only present in the second multithreading run. In the second dataset, 3,664 variants (2%) are only present in the first run, while 55 variants are only present in the second run.

The largest number of differences are seen with GATK. In the first dataset, 1,466 out of 1,650 (89%) total calls on target differ in the “QUAL”-value and/or the information provided in the “INFO” filed regarding the reported VCF files. Additionally, 126 variants are only reported in the first run, while 146 variants are only reported in the second multithreading run. In the second dataset, 1,188 out of 1,559 (76%) total calls on target show differences concerning the “QUAL”-value and/or information provided in the “INFO” field. 115 variants are only reported in the first run, while 126 variants are only reported in the second multithreading run. GATK does not succeed in reporting an exactly identical output for a single sample.

### Output comparability

GATK, Platypus, LoFreq, FreeBayes and SAMtools all report one VCF file per sample. Apart from basic information on the called variants, e.g. the chromosomal position, all files contain a specific set of reported values, including various tests and flags that differs considerably between different tools (see Supplementary Table S13 for details). SNVer reports four VCF files per sample – two files containing raw SNVs and indels and two files containing the automatically filtered SNVs and indels. VarDict reports one TXT file containing basic and additional information on the calls. VarScan reports two TXT files – one concerning SNVs and one concerning indels.

There are considerable differences between the different variant calling tools regarding the way insertions and deletions are reported. The exemplary mutation *chr2:25456668 delA* is reported by GATK, Platypus, LoFreq, SNVer and VarDict as “2 25456667 CA C” and by VarScan as “2 25456667 C -A”. FreeBayes and SAMtools however report the surrounding bases if the insertion or deletion is affecting a repetitive region. In case of the example “2 25456667CAAAAAAAAAAAACCAAAAAAAAAAAC” is reported. The repetitive regions are not limited to homopolymers, but also cover complex repeats.

An additional matter is multi-nucleotide variants (MNVs; variants that involve more than one nucleotide). If two or more mutations are detected in close proximity, FreeBayes and VarDict report them as MNVs, whereas all the other tools report distinct mutations.

A comprehensive combined evaluation of the different variant calling tools’ output would therefore require normalization (e.g. with the help of “vt normalize”^24^) of the output of every variant calling tool.

### Run time

All variant calling tools can be easily downloaded and installed. Analysis of the 54 Illumina HiSeq samples (first dataset) and the 111 Illumina NextSeq samples (second dataset) is performed on our server (256 GB RAM, Intel Xeon CPU with 2.60 GHz, 1 core). The run time for variant calling, performed as displayed in Supplementary Table S11, is summed up in Table 2.

All variant calling tools differ considerably with respect to run time. The fastest tool in case of the first dataset is Platypus, completing the analysis of the 54 samples in less than one hour. In contrast, GATK is the slowest tool, consuming more than 17 hours for the analysis. For the second dataset, SAMtools is the fastest tool, while SNVer is the slowest.

The second dataset sequenced on NextSeq contains double as many samples as the first dataset (HiSeq). Furthermore, the second dataset contains 78-times as many total reads on target as the first dataset (3 billion compared to 38 million). Remarkably, we find that all tools but Platypus show a considerably smaller increase in run time than 78-fold. For example, VarScan shows an increase by a factor of 4.5 only.

The influence of coverages on run time can also be investigated by using the simulated datasets. Supplementary Figure S4 in general shows a linear increase in run time. SNVer shows a larger increase than expected in case of a linear correlation. For GATK a linear increase in run time is only observed until 4,000x coverage. Simulation of 6,000x coverage leads to a larger increase in run time in case of SIM1 (HiSeq), but a weaker increase in case of SIM2 (NextSeq).

It is expected that the level of background noise influences run time as well. Supplementary Figures S5 and S6 indicates that increased background noise indeed increases run time. The increase is most obvious in case of GATK, FreeBayes and VarDict analysing simulated HiSeq data.

Regarding run time it has to be taken into account, that GATK as well as VarScan, LoFreq and SAMtools contain pre-processing steps, which increase the run time. However, as these steps are either necessary or highly recommended by the authors of the tools, they are regarded as being an integral part of the variant calling process and thus contribute to the reported CPU time.

## Discussion

Next-generation sequencing may be regarded as one of the key-factors influencing the ongoing development of personalized medicine. There are plenty of tools for performing variant calling of SNVs and indels in NGS data. Considering two real and two simulated datasets, we evaluated eight variant calling tools – GATK, Platypus, VarScan, LoFreq, FreeBayes, SNVer, SAMtools and VarDict – to identify the momentary possibilities and limits of mutational screening by NGS.

Combining our results on sensitivity in case of real- and simulated data, confident calling of low- and high frequency variants can best be achieved by using FreeBayes or VarDict. Use of LoFreq and Platypus is, according to our analysis, only safe for calling variants present with *VAFs* > 0.10. Sensitivity of GATK seemed to be highly data-dependent. Therefore, we would not suggest to use GATK to detect variants with *VAF* ≤ 0.20. These results are in line with the Broad Institute’s recommendation not to use GATK for detecting variants with extreme allele frequencies^14^. Same is true for SAMtools and VarScan. Regarding SNVer, we observed a very unstable performance. As no clear tendency towards higher sensitivity with increasing allelic frequencies could be observed, we cannot define a VAF threshold above which SNVer could be used to confidently call variants.

With respect to precision, data clearly show that high PPVs are not achievable with any variant calling tool when variants are present at low frequencies and no advanced steps of post-processing are applied. If *VAF* ≤ 0.05, PPV of all variant calling tools is usually close to zero. The only exceptions from this observation are VarDict and SNVer in case of the Illumina HiSeq datasets. A data-dependent increase in PPV can be observed as VAF increases. Taking all data into account, VarDict features the best performance. A relatively stable *PPV* > 0.80 can be observed if *VAF* > 0.15.

Combining our results on sensitivity and PPV, VarDict performs best on all datasets we analysed. Although the variant caller does not succeed in calling all mutations present, it does succeed in calling a majority of variants – even with very low frequencies. Furthermore, VarDict usually features high PPV even at low allelic frequencies and is relatively invariant towards varying coverage and varying background noise.

It can be argued that we did not perform any caller-specific filtration steps. It is likely that PPV could have been improved, if the default options had been changed and filtration of caller-specific parameters had been performed. However, no tool provides any recommendations on how to change the default options if dealing with a certain type of data. Investigating all possible combinations and their influence on the results is practically impossible. Furthermore, all tools report certain parameters characterizing their reported variants, but GATK is the only tool proposing concrete thresholds that could be used for filtration. As this would, in our opinion, result in a biased processing of reported calls, we decided to rely on our filters only – treating all calls equally.

The evaluation we perform is based on a correct classification of the variants being reported. However, – given the large number of variants that were called (in total 46,535; i.e. on average more than 282 per patient) – we did not validate all variants reported by all different variant calling tools. We therefore cannot exclude the possibility that some true mutations – especially those with low allelic frequencies – might have been classified as artefacts. Nevertheless, we have shown that our classification was indeed correct, considering an exemplary subset of mutations, artefacts and polymorphisms. We use sequencing results of Sanger sequencing, sequencing on different platforms and sequencing on the same platform for validation. All the other calls were classified based on biological and bioinformatical expert opinion, taking into account the presence of variants in various databases, the mean base qualities, the coverage, the allelic frequencies and the predicted effect on protein level. A high percentage of calls was even inspected manually, considering the sequencing context within the same sample and other samples in the same run. We therefore assume that the chance of having classified some variants incorrectly is low and not expected to influence the overall results.

When comparing real- and simulated datasets, it becomes obvious that data show considerable differences with respect to PPV. This observation limits transferability of the simulated results. We investigate, whether our observation might due to problems in the simulation of the error profile. For HiSeq data, *noise*_*real*_ = 5.39 · 10^−3^, while *noise*_*sim*_ = 1.81 · 10^−2^. Consequently, we would expect worse instead of better results regarding PPV in case of the simulated HiSeq data. With NextSeq data, *noise*_*real*_ = 6.26 · 10^−3^ and *noise*_*sim*_ = 1.85 · 10^−3^. Thus, the observed better PPVs in case of the simulated data might be due to problems in the simulation of the NextSeq error profile.

In addition, we consider the real and simulated artefacts themselves. Comparing Supplementary Figs 7a and 7c it becomes obvious that some simulated sequencing errors are comparable to real sequencing errors. However, common artefacts in real data as displayed in Fig. 7b (strand bias, read position bias, called in 50–100% of all samples) are not present in any of the simulated datasets. Furthermore, an increase in sequencing errors in the context of homopolymeric or other repetitive regions that can clearly be observed in real data (Supplementary Fig. 8a), cannot be detect in simulated data – not even in case of doubled error frequencies (Supplementary Fig. 8b). Thus, a lack of realistically simulated sequencing errors is likely to have huge influence on the observed discrepancies in PPV.

In case of both the simulation of varying coverages and varying background noise we observed unexpected results. Regarding the fact that simulated data differ from real data, it appears possible that these unexpected results are partly due to the simulated data itself. Especially in the case of varying background noise, a realistic simulation of systematic and random sequencing errors is essential. However, background noise in case of the simulated HiSeq data indicates that simulation has not been thoroughly successful. Regarding simulation with doubled error rates, an increase in background noise of only 10% can be observed. It thus seems possible that simulated data with a higher level of background noise might have led to different results. Real sequencing data with predetermined levels of background noise would have been useful. However, as this is impossible, we had to rely on the simulated data.

In conclusion, we observe a rapid and ongoing increase in the use of next-generation sequencing. However, variant calling – even of SNVs and short indels – remains challenging. Our evaluation of eight open-source variant calling tools on two different real- and simulated datasets pointed out that perfect results could not be obtained with any of the tools.

The best results with respect to sensitivity and PPV can currently be achieved by using VarDict. Our observation that different true mutations are missed by different tools indicates that a considerable improvement in the results might be achieved by normalizing and combining the output of different tools. We are currently working on an automatic variant calling pipeline, realizing this approach.

## Methods

### Tools investigated

To evaluate variant calling tools for next-generation sequencing data, we looked for open-source tools with the ability of calling SNVs and short indels. We focused our search on PubMed, using the keyword “variant call*”. We equally considered articles describing new tools, as well as review articles (publication type “review”) comparing common variant calling tools. In addition, we analysed the references of the papers and considered expert opinions on variant calling.

Altogether, our search identified 43 open-source tools performing variant calling in NGS data. However, 35 variant callers were excluded due to different reasons (e.g. matched samples required, tool unavailable, no new variant calling algorithm) and only eight tools were considered for further evaluation. An overview of the tools and the exclusion criteria can be found in Supplementary Table S10.

The eight open-source variant calling tools we investigated are: GATK 3.3-0 HaplotypeCaller^14^, Platypus 0.8.1^15^, VarScan 2.3.9^18^, LoFreq 2.1.2^25^, FreeBayes 1.0.2^16^, SNVer 0.5.3^19^, SAMtools 1.3^17^ and VarDict^20^. Every variant caller contains numerous options for individual calibration. To allow a fair comparison of the eight tools, we chose to stick to the default recommended options for every variant caller (see Supplementary Table S11 for the exact command lines). The only exception was the variant allele frequency (VAF) threshold in case of FreeBayes and SNVer, which was lowered to 0.01 (default 0.20, resp. 0.25).

Regarding VarScan, LoFreq and SAMtools it was necessary to perform steps of pre-processing. In case of GATK, recalibration of the base qualities is recommended and was thus performed prior to the actual variant calling.

### Datasets analysed

Two sets of real amplicon-based targeted sequencing data resulting from MDS patients were investigated. In case of both sets, 19 genes, which are known to be recurrently mutated in MDS, were analysed (see Supplementary Table S12 for detailed information on the target region). Altogether, the target region covered 42,322 bp. The first set contains data from 54 samples sequenced on Illumina HiSeq, using HaloPlex target enrichment kit (Agilent Inc., on average 1,983x coverage; background noise ≤0.54%; all samples were collected and analyzed according ethical guidelines approved by Karolinska Institutet ethical committee; methods were carried out in accordance with the relevant guidelines and regulations; informed consent was obtained from all subjects). The second set contains data from 111 samples sequenced on Illumina NextSeq, using TruSight DNA Amplicon Sequencing Panel (Illumina Inc., on average 4,034x coverage; background noise ≤0.63%; the study was approved by MEC: Medisch Ethische Toetsingscommissie of the Vrije Universiteit Medisch Centrum; methods were carried out in accordance with the relevant guidelines and regulations; all participants provided written consent to participate in this study; EudraCT nr.: 2008-002195-10; date of registration: May, 19th 2009).

We decided to choose these datasets for evaluating the eight variant calling tools, as they both consist of a high number of samples, sequenced with the latest technologies. The analysed samples form a homogeneous group as they all result from MDS patients. The same target region is sequenced in all cases. Furthermore, the datasets are well characterized with respect to biological truth.

In addition, we considered two sets of simulated targeted sequencing data (using ART_Illumina 2.5.8^26^) containing simulated mutations and polymorphisms (using bam surgeon^27^). The first simulated set covers 50 samples of Illumina HiSeq data. The second simulated set covers 50 samples of Illumina NextSeq data. To investigate the influence of varying coverage and background noise on the performance of the different variant calling tools, we simulated seven different scenarios per simulated dataset, which are summed up in Table 3. In order to assess the negative predictive value (NPV), we simulated two additional sets covering 50 samples each, containing polymorphisms that have not been detected in any of the real samples (for details see Supplementary Sections S1.2 and S2.2).

Based on the real data, read quality profiles are determined – one for the HiSeq data and one for the NextSeq data. These profiles form the basis for the simulation in order to obtain simulated data, that are very similar to the original. Furthermore, coverages are chosen that are identical to the original data (SIM1_E1_C3 and SIM2_E1_C2).

To investigate the influence of varying coverage, five different levels of coverage are considered, including coverage of the original data (SIM1_E1_C1-SIM1_E1_C5 and SIM2_E1_C1-SIM2_E1_C5). To investigate the influence of varying background noise on the results, the same simulated datasets with doubled (SIM1_E2_C3 and SIM2_E2_C2) as well as half error rates (SIM1_E3_C3 and SIM2_E3_C2) are considered. Details on the simulation can be found in Supplementary Section S1.

### Analysis pipeline

**Figure 5:**
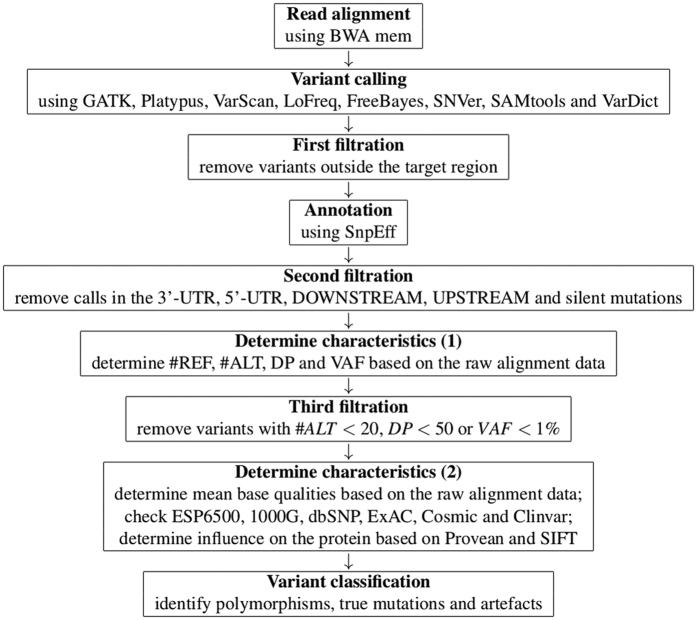
Overview of the analysis pipeline.

An overview of the analysis pipeline is provided in Fig. 5.

Read alignment was in case of both datasets performed using BWA mem^28^. The resulting BAM files formed the input for the eight variant callers. Every tool processed exactly the same files. As only calls on target were of interest, all calls outside the target region were removed from the raw output (first filtration).

Annotation of the remaining calls was performed using SNPeff 29. As NGS analysis in the use-case of clinical routine mainly focuses on variants in the coding region, we excluded all calls in the non-coding region from further analysis. All calls located in the 3′-UTR, 5′-UTR, downstream and upstream were filtered from the list. In addition, silent mutations were excluded (second filtration).

For the remaining calls, the number of reads containing the reference allele (#REF), the number of reads containing the alternate allele (#ALT), the depth (DP) and the variant allele frequency (VAF) was determined using R^30^ (http://www.R-project.org). Some variant callers, like GATK or VarDict, automatically perform local realignment when calling variants. As this rearrangement of the reads can lead to changes in #REF, #ALT, DP and VAF, the raw BAM files resulting from the alignment were used to determine these characteristics. All variants with #*ALT* < 20, *DP* < 50 or *VAF* < 1% were removed afterwards (3rd filtration).

A second set of characteristics was determined for the remaining calls. Mean base qualities for reference- and alternate alleles were determined. An automatic check of the databases ESP6500^31^, 1000G^32^, dbSNP^33^ (build 138, referred to as “dbSNP” and build 138 excluding sites after 129, referred to as “dbSNP (polymorphisms only)”), ExAC^34^, Cosmic^35^ (CodingMuts and NonCodingVariants, 17.02.2016) and ClinVar^36^ (common and clinical, 03.02.2016; common no known medical impact, 03.02.2016) was performed. Additionally, the influence of every variant on the corresponding protein was determined based on Provean 1.1.3 and SIFT^37^. Based on this information, the remaining calls were categorized as polymorphisms, true mutations and artefacts.

#### Variant classification

For the identification of polymorphisms and true mutations in the set of called variants, a combination of various criteria is evaluated by biological experts: knowledge of a variant being an MDS-related hotspot mutation, presence and frequency of a variant in ESP6500, 1000G, ExAC, Cosmic, Clinvar (common, no medical impact), ClinVar (common and clinical), dbSNP (polymorphisms only), dbSNP, presence of a *PM* flag (precious variant), variant allele frequency, mean base qualities, effect of the variant on the protein based on Provean and SIFT and the number of samples that feature the same call.

Assuming that none of the criteria mentioned above is free from flaws, we always considered a combination to identify polymorphisms and true mutations. If a variant had a VAF close to 0.5 or 1.0, if it was present in one of the polymorphism databases (ESP6500, 1000G, ExAC, ClinVar “common, no medical impact” and dbSNP (polymorphisms only)), if the alternate allele had a high mean base quality and/or the variant had a neutral effect on the protein, it was regarded as a candidate for being a polymorphism. However, the final decision was always made individually for every call by two independent biological experts, taking into account all the available information.

If a variant was a known hotspot mutation, it was regarded as a candidate for being a true mutation. In addition, a variant was considered being a potentially true mutation if its VAF was not close to 0.5 or 1.0, if it was present in one of the mutation databases (Cosmic, ClinVar “common and clinical”, dbSNP), if it was not present in any of the polymorphism databases, if the alternate allele had a high mean base quality and/or if the variant had a damaging effect on the protein. All those candidates for being true mutations were investigated in the IGV^38^ as well as neighbouring samples. A final decision was made individually for every call by two independent biological experts, taking into account all the available information. A classification of a variant as a mutation and a polymorphism at the same time is not possible.

A called variant was considered being a potential artefact if it was present in many samples, the VAFs were not close to 0.5 or 1.0, the variant was located in a repetitive region, the alignment was bad, the mean base quality of the alternate allele was low compared to the reference allele and/or the variant was not present in any of the considered databases. If a call could not be identified as an obvious artefact (e.g. mean base quality of the alternate allele *BQ*_*alt* = 5 in comparison to the reference allele *BQ*_*ref* = 32), it was manually inspected by the help of the IGV.

In addition, artefacts due to leakage were considered. If a called variant featured a relatively high quality, but a low VAF, the identified true mutations and polymorphisms were checked for the call. If the same variant was called for a neighbouring sample (i.e. a sample in close physical proximity during sequencing) with a high VAF, the call with the low VAF was classified as an artefact.

#### Validation of the classification

To validate our protocol for identifying polymorphisms, mutations and artefacts, a selected set of calls (polymorphisms, mutations and artefacts) in both datasets was validated using Sanger sequencing. However, as the confirmation of variants with VAFs below 20% is not possible with this sequencing technique, we re-analysed a subset of six samples on the same sequencer (Illumina NextSeq). In addition, we re-analysed a subset of samples on different sequencers, as we assume that different sequencing techniques will report different artefacts, but the same true mutations and polymorphisms. Twenty-two samples were re-analysed on Roche 454^1^ and nine samples were re-analysed on Ion Torrent PGM^39^. Furthermore, expert based review of all calls was performed by two independent experts.

## Additional Information

**How to cite this article:** Sandmann, S. *et al*. Evaluating Variant Calling Tools for Non-Matched Next-Generation Sequencing Data. *Sci. Rep.* **7**, 43169; doi: 10.1038/srep43169 (2017).

**Publisher's note:** Springer Nature remains neutral with regard to jurisdictional claims in published maps and institutional affiliations.

## Supplementary Material

Supplementary Information

Supplementary Dataset 1 - Mutations

Supplementary Dataset 1 - Polymorphisms

Supplementary Dataset 1 - Artifacts

Supplementary Dataset 2 - Mutations

Supplementary Dataset 2 - Polymorphisms

Supplementary Dataset 2 - Artifacts

## Figures and Tables

**Table 1:** Number of called mutations (number of SNVs and indels given in brackets) and artefacts, sensitivity (sens), positive predictive value (PPV) and the *F*_1_ score regarding the eight variant calling tools investigated in case of the first and second dataset.

Variant caller	First real dataset (HiSeq)					Second real dataset (NextSeq)				
	Mutations (SNVs + indels)	Artefacts	Sens	PPV	*F*_1_ score	Mutations (SNVs + indels)	Artefacts	Sens	PPV	*F*_1_ score
GATK	94 (76 + 18)	55	0.83	0.63	0.72	156 (112 + 44)	209	0.69	0.43	0.53
Platypus	95 (76 + 19)	67	0.84	0.58	0.69	189 (136 + 53)	791	0.83	0.19	0.31
VarScan	85 (69 + 15)	7	0.75	0.92	0.83	91 (84 + 7)	109	0.40	0.46	0.43
LoFreq	111 (91 + 20)	503	0.98	0.18	0.31	178 (121 + 57)	1,650	0.79	0.10	0.17
FreeBayes	113 (93 + 20)	7,918	1.00	0.01	0.03	178 (162 + 16)	35,126	0.79	0.01	0.01
SNVer	94 (76 + 18)	24	0.83	0.80	0.81	127 (72 + 55)	4,389	0.56	0.03	0.05
SAMtools	72 (70 + 2)	37	0.64	0.65	0.65	73 (71 + 2)	111	0.32	0.40	0.36
VarDict	109 (90 + 19)	9	0.96	0.92	0.94	205 (155 + 50)	3,517	0.91	0.06	0.10
Ground truth	113 (93 + 20)	0				226 (168 + 58)	0			

**Table 2:** Run time of the variant calling process for the eight variant calling tools investigated regarding the first and second dataset.

Variant caller	Preprocessing	CPU time (first dataset)	CPU time (second dataset)	Option for multithreading
GATK	yes	1,021 min	4,876 min	yes
Platypus	no	59 min	9,952 min	yes
VarScan	yes	649 min	2,906 min	no
LoFreq	yes	945 min	11,991 min	yes
FreeBayes	no	732 min	6,652 min	no
SNVer	no	492 min	14,431 min	no
SAMtools	yes	305 min	1,859 min	no
VarDict	no	281 min	3,513 min	no

**Table 3:** Simulated scenarios for the simulated Illumina HiSeq and NextSeq datasets.

Sequencer	Error rate	Cov = 6,000x	Cov = 4,034x	Cov = 1,983x	Cov = 1,000x	Cov = 500x
HiSeq	HiSeq	SIM1_E1_C1	SIM1_E1_C2	**SIM1**_**E1**_**C3**	SIM1_E1_C4	SIM1_E1_C5
	HiSeq*2			SIM1_E2_C3		
	HiSeq/2			SIM1_E3_C3		
	HiSeq			SIM1_E1_C3_NPV		
NextSeq	NextSeq	SIM2_E1_C1	**SIM2**_**E1**_**C2**	SIM2_E1_C3	SIM2_E1_C4	SIM2_E1_C5
	NextSeq*2		SIM2_E2_C2			
	NextSeq/2		SIM2_E3_C2			
	NextSeq		SIM2_E1_C2_NPV			
