# Supplementary material for: Evaluating Variant Calling Tools for Non-Matched Next-Generation Sequencing Data
Source: Sci Rep. 2017 Feb 24;7:43169. doi: 10.1038/srep43169 (PMC5324109; doi:10.1038/srep43169)
Supplement: Supplementary Information [file srep43169-s1.pdf]

Supplement: Evaluating Variant Calling Tools for Non-Matched Next-Generation Sequencing Data

Sarah Sandmann[1,*], Aniek O de Graaf[2], Mohsen Karimi[3], Bert A van der Reijden[2], Eva Hellström-Lindberg[3], Joop H Jansen[2], Martin Dugas[1]

[1]Institute of Medical Informatics, University of Münster, Münster, 48149, Germany
[2]Laboratory Hematology, RadboudUMC, Nijmegen, 6525, Netherlands
[3]Center for Hematology and Regenerative Medicine, Department of Medicine Huddinge, Karolinska Institutet, Stockholm, 14186, Sweden
*sarah.sandmann@uni-muenster.de

1 Simulated data

1.1 Simulation of the raw sequences

Simulation of the raw sequences was performed using ART_Illumina 2.5.8[18]. Prior to the actual simulation of the reads, read quality profiles characterizing the original Illumina HiSeq, resp. Illumina NextSeq data were determined:

```
./art_profiler_illumina hiseq $DIR/seqs fastq
./art_profiler_illumina nextseq $DIR/seqs fastq
```

Subsequently, the actual raw sequences were simulated:

```
SIM1_E1_C1   ./art_illumina -ss HS20 -sam -i $REF -l 100 -f 6000
             -1 $DIR/Error/hiseq.txt -rs $START -na -M
             -o $DIR/alignment/$SAMPLE_tmp
SIM1_E1_C2   ./art_illumina -ss HS20 -sam -i $REF -l 100 -f 4034
             -1 $DIR/Error/hiseq.txt -rs $START -na -M
             -o $DIR/alignment/$SAMPLE_tmp
SIM1_E1_C3   ./art_illumina -ss HS20 -sam -i $REF -l 100 -f 1983
             -1 $DIR/Error/hiseq.txt -rs $START -na -M
             -o $DIR/alignment/$SAMPLE_tmp
SIM1_E1_C4   ./art_illumina -ss HS20 -sam -i $REF -l 100 -f 1000
             -1 $DIR/Error/hiseq.txt -rs $START -na -M
             -o $DIR/alignment/$SAMPLE_tmp
SIM1_E1_C5   ./art_illumina -ss HS20 -sam -i $REF -l 100 -f 500
             -1 $DIR/Error/hiseq.txt -rs $START -na -M
             -o $DIR/alignment/$SAMPLE_tmp
SIM1_E2_C3   ./art_illumina -ss HS20 -sam -i $REF -l 100 -f 1983
             -1 $DIR/Error/hiseq.txt -qs -3.0103 -rs $START -na -M
             -o $DIR/alignment/$SAMPLE_tmp
SIM1_E3_C3   ./art_illumina -ss HS20 -sam -i $REF -l 100 -f 1983
             -1 $DIR/Error/hiseq.txt -qs 3.0103 -rs $START -na -M
             -o $DIR/alignment/$SAMPLE_tmp
SIM2_E1_C1   ./art_illumina -ss NS50 -sam -i $REF -l 75 -f 6000
             -1 $DIR/Error/nextseq.txt -rs $START -na -M
             -o $DIR/alignment/$SAMPLE_tmp
SIM2_E1_C2   ./art_illumina -ss NS50 -sam -i $REF -l 75 -f 4034
             -1 $DIR/Error/nextseq.txt -rs $START -na -M
             -o $DIR/alignment/$SAMPLE_tmp
SIM2_E1_C3   ./art_illumina -ss NS50 -sam -i $REF -l 75 -f 1983
             -1 $DIR/Error/nextseq.txt -rs $START -na -M
             -o $DIR/alignment/$SAMPLE_tmp
SIM2_E1_C4   ./art_illumina -ss NS50 -sam -i $REF -l 75 -f 1000
             -1 $DIR/Error/nextseq.txt -rs $START -na -M
             -o $DIR/alignment/$SAMPLE_tmp
```

SIM2_E1_C5	`./art_illumina -ss NS50 -sam -i $REF -l 75 -f 500 -1 $DIR/Error/nextseq.txt -rs $START -na -M -o $DIR/alignment/$SAMPLE_tmp`
SIM2_E2_C2	`./art_illumina -ss NS50 -sam -i $REF -l 75 -f 4034 -1 $DIR/Error/nextseq.txt -qs -3.0103 -rs $START -na -M -o $DIR/alignment/$SAMPLE_tmp`
SIM2_E3_C2	`./art_illumina -ss NS50 -sam -i $REF -l 75 -f 4034 -1 $DIR/Error/nextseq.txt -qs 3.0103 -rs $START -na -M -o $DIR/alignment/$SAMPLE_tmp`

The simulation produces fastq-files, which are aligned to the reference genome (GRCh37.67) using BWA mem[22].

1.2 Simulation of the polymorphisms and mutations

For the simulation of the polymorphisms and mutations two lists, based on the real datasets, are defined. The first list contains a selection of ten common polymorphisms (see table 1) that have been detected in the real datasets. The polymorphisms have been chosen to cover as many different genes as possible and to consider SNPs as well as indel polymorphisms.

Table 1. List of selected, common polymorphisms present in the real datasets.

chr	pos	ref	alt
2	25523096	T	G
4	106196951	A	G
7	148525904	C	G
11	119149352	A	G
12	12022496	T	C
17	7579472	G	C
19	33792731	G	GGCGGGT
20	31022959	T	C
21	36259324	A	G
X	15841230	C	CAGCCGG

In order to asses the negative predictive value, we also considered a set of ten common polymorphisms that have not been called in any of the real samples.

Table 2. List of selected, common polymorphisms absent in the real datasets.

chr	pos	ref	alt
1	115256457	T	C
1	115258744	C	T
1	115258755	AACC	A
2	25463286	C	T
2	25463265	G	A
4	106196222	G	A
9	5069973	C	T
12	12022390	G	A
13	28592684	G	A
21	36206814	C	T

The third list contains fifteen hotspot mutations and their mean observed frequencies (see table 3). All mutations on the list have been detected in the analyzed real datasets and are frequently associated with MDS. The mutations have been chosen to cover the most common hotspot mutations associated with MDS, to cover different genes in the target region, to consider high- as well as low-frequency mutations and to consider SNVs as well as indels.

Using R[27] (http://www.R-project.org) the number of polymorphisms and mutations that shall be added to the simulated raw sequencing data is determined. As we are aiming at simulating data that are most similar to the real data, we use the characteristics of the real dataset as a basis. The number of polymorphisms and mutations in the Illumina HiSeq dataset (mean

Table 3. List of selected hotspot mutations and their observed frequencies present in the real datasets.

chr	pos	ref	alt	freq
1	115258744	C	A	0.03
1	115258747	C	T	0.03
2	198266834	T	C	0.30
2	198267371	G	T	0.30
2	198267484	G	A	0.25
4	106164778	C	T	0.30
7	148523591	G	A	0.50
9	5073770	G	T	0.03
15	90631934	C	T	0.20
17	7579314	T	G	0.90
17	74732935	CGGCGGCTGTGGTGTGAGTCCGGGG	C	0.35
20	31022402	TCACCACTGCCATAGAGAGGCGGC	T	0.08
20	31022441	A	AG	0.25
21	36231782	C	T	0.30
21	44524456	G	T	0.20

and standard deviation) is determined. Subsequently, for every simulated HiSeq sample, a random number of polymorphisms and mutations is determined on the basis of the corresponding mean and standard deviation. Analogously, the number of polymorphisms and mutations for the simulated NextSeq samples are determined.

We assume that the presence of one polymorphisms and mutations has no influence on the presence or absence of another polymorphisms and mutations. Consequently, every variant has the same chance of being added to the simulated raw data. We use a uniform distribution to randomly select the polymorphisms and mutations that shall be inserted for ever simulated sample.

Considering polymorphisms, we assume that it is equally likely for a polymorphism being homozygous or heterozygous. To take random variation in the observed frequencies – 0.5 in case of heterozygous polymorphisms, 1.0 in case of homozygous polymorphisms – into account, a normal distribution with $N(\mu, \mu \cdot 0.2)$ is used.

Regarding mutations, we use the expected frequencies as a basis for the simulation. To take random variation in these frequencies in to account, a normal distribution with $N(\mu, \mu \cdot 0.2)$ is once again used to determine the frequencies that shall be used for the simulation.

Thus, for every simulated sample, an individual set of polymorphisms and mutations with individual frequencies exists. However, the same sample in a different scenario, e.g. sample 01 in SIM1_E1_C1 and SIM1_E1_C2, features the same polymorphisms and mutations with the same frequencies. This approach allows for a direct analysis of the influence of varying coverage and background noise on the variant calling results.

The actual simulation of the polymorphisms and mutations is performed using bam surgeon[13]. SNVs, indels, SNPs and indel polymorphisms are successively added to the simulated raw sequencing data:

SNVs:

```
cp $DIR/alignment/$SAMPLE.bai $DIR/alignment/$SAMPLE.bam.bai
if [ -s $DIR/Mutations/$SAMPLE_snv.bed ]; then
  python ./addsnv.py -v $DIR/Mutations/$SAMPLE_snv.bed
  -f $DIR/alignment/$SAMPLE.bam -r $GENOME
  -o $DIR/Mutated/$SAMPLE_snv_temp1.bam --seed $START --single
  --picardjar $PICARD/picard.jar --aligner mem --maxdepth 12000
  samtools view -h $DIR/Mutated/$SAMPLE_snv_temp1.bam | awk '{OFS="\t";
  print $1,$2,$3,$4,$5,$6,$7,$8,$9,$10,$11}'
  > $DIR/Mutated/$SAMPLE_snv_temp2.sam
  samtools view -h $DIR/Mutated/$SAMPLE_snv_temp2.sam | tail -n+2
  > $DIR/Mutated/$SAMPLE_snv_temp3.sam
  java -jar $PICARD/picard.jar AddOrReplaceReadGroups \
    I=$DIR/Mutated/$SAMPLE_snv_temp3.sam \
    O=$DIR/Mutated/$SAMPLE_snv_temp4.bam \
    SORT_ORDER=coordinate \
    CREATE_INDEX=true \
    VALIDATION_STRINGENCY=LENIENT \
    RGID=1 \
    RGLB=JULY2016 \
    RGPL=ILLUMINA \
    RGPU=NA \
    RGSM=$SAMPLE
  ((START=START+1))
else
  cp $DIR/alignment/$SAMPLE.bam $DIR/Mutated/$SAMPLE_snv_temp4.bam
  cp $DIR/alignment/$SAMPLE.bam.bai $DIR/Mutated/$SAMPLE_snv_temp4.bai
fi
```

Indels:

```
cp $DIR/Mutated/$SAMPLE_snv_temp4.bai $DIR/Mutated/$SAMPLE_snv_temp4.bam.bai
if [ -s $DIR/Mutations/$SAMPLE_indel.bed ]; then
  python ./addindel.py -v $DIR/Mutations/$SAMPLE_indel.bed
  -f $DIR/Mutated/$SAMPLE_snv_temp4.bam -r $GENOME
  -o $DIR/Mutated/$SAMPLE_indel_temp1.bam --seed $START --single
  --picardjar $PICARD/picard.jar --aligner mem --maxdepth 12000
  samtools view -h $DIR/Mutated/$SAMPLE_indel_temp1.bam | awk '{OFS="\t";
  print $1,$2,$3,$4,$5,$6,$7,$8,$9,$10,$11}'
  > $DIR/Mutated/$SAMPLE_indel_temp2.sam
  samtools view -h $DIR/Mutated/$SAMPLE_indel_temp2.sam | tail -n+2
  > $DIR/Mutated/$SAMPLE_indel_temp3.sam
  java -jar $PICARD/picard.jar AddOrReplaceReadGroups \
    I=$DIR/Mutated/$SAMPLE_indel_temp3.sam \
    O=$DIR/Mutated/$SAMPLE_indel_temp4.bam \
    SORT_ORDER=coordinate \
    CREATE_INDEX=true \
    VALIDATION_STRINGENCY=LENIENT \
    RGID=1 \
    RGLB=JULY2016 \
    RGPL=ILLUMINA \
    RGPU=NA \
    RGSM=$SAMPLE
  ((START=START+1))
else
  cp $DIR/Mutated/$SAMPLE_snv_temp4.bam
  $DIR/Mutated/$SAMPLE_indel_temp4.bam
  cp $DIR/Mutated/$SAMPLE_snv_temp4.bam.bai
  $DIR/Mutated/$SAMPLE_indel_temp4.bai
fi
```

SNPs:

```
cp $DIR/Mutated/$SAMPLE_indel_temp4.bai $DIR/Mutated/$SAMPLE_indel_temp4.bam.bai
if [ -s $DIR/Polymorphisms/$SAMPLE_snp.bed ]; then
  python ./addsnv.py -v $DIR/Polymorphisms/$SAMPLE_snp.bed
  -f $DIR/Mutated/$SAMPLE_indel_temp4.bam -r $GENOME
  -o $DIR/Mutated/$SAMPLE_snp_temp1.bam --seed $START --single
  --picardjar $PICARD/picard.jar --aligner mem --maxdepth 12000
  samtools view -h $DIR/Mutated/$SAMPLE_snp_temp1.bam | awk '{OFS="\t";
  print $1,$2,$3,$4,$5,$6,$7,$8,$9,$10,$11}'
  > $DIR/Mutated/$SAMPLE_snp_temp2.sam
  samtools view -h $DIR/Mutated/$SAMPLE_snp_temp2.sam | tail -n+2
  > $DIR/Mutated/$SAMPLE_snp_temp3.sam
  java -jar $PICARD/picard.jar AddOrReplaceReadGroups \
    I=$DIR/Mutated/$SAMPLE_snp_temp3.sam \
    O=$DIR/Mutated/$SAMPLE_snp_temp4.bam \
    SORT_ORDER=coordinate \
    CREATE_INDEX=true \
    VALIDATION_STRINGENCY=LENIENT \
    RGID=1 \
    RGLB=JULY2016 \
```

```
      RGPL=ILLUMINA \
      RGPU=NA \
      RGSM=$SAMPLE
    ((START=START+1))
  else
    cp $DIR/Mutated/$SAMPLE_indel_temp4.bam
    $DIR/Mutated/$SAMPLE_snp_temp4.bam
    cp $DIR/Mutated/$SAMPLE_indel_temp4.bam.bai
    $DIR/Mutated/$SAMPLE_snp_temp4.bai
  fi
```

Indel polymorphisms:

```
cp $DIR/Mutated/$SAMPLE_snp_temp4.bai $DIR/Mutated/$SAMPLE_snp_temp4.bam.bai
if [ -s $DIR/Polymorphisms/$SAMPLE_indelp.bed ]; then
  python ./addindel.py -v $DIR/Polymorphisms/$SAMPLE_indelp.bed
  -f $DIR/Mutated/$SAMPLE_snp_temp4.bam -r $GENOME
  -o $DIR/Mutated/$SAMPLE_indelp_temp1.bam --seed $START --single
  --picardjar $PICARD/picard.jar --aligner mem --maxdepth 12000
  samtools view -h $DIR/Mutated/$SAMPLE_snp_temp1.bam | awk '{OFS="\t";
  print $1,$2,$3,$4,$5,$6,$7,$8,$9,$10,$11}'
  > $DIR/Mutated/$SAMPLE_indelp_temp2.sam
  samtools view -h $DIR/Mutated/$SAMPLE_indelp_temp2.sam | tail -n+2
  > $DIR/Mutated/$SAMPLE_indelp_temp3.sam
  java -jar $PICARD/picard.jar AddOrReplaceReadGroups \
    I=$DIR/Mutated/$SAMPLE_indelp_temp3.sam \
    O=$DIR/Mutated/$SAMPLE_indelp_temp4.bam \
    SORT_ORDER=coordinate \
    CREATE_INDEX=true \
    VALIDATION_STRINGENCY=LENIENT \
    RGID=1 \
    RGLB=JULY2016 \
    RGPL=ILLUMINA \
    RGPU=NA \
    RGSM=$SAMPLE
  ((START=START+1))
else
  cp $DIR/Mutated/$SAMPLE_snp_temp4.bam
  $DIR/Mutated/$SAMPLE_indelp_temp4.bam
  cp $DIR/Mutated/$SAMPLE_snp_temp4.bam.bai
  $DIR/Mutated/$SAMPLE_indelp_temp4.bai
fi
```

The resulting simulated samples are directly analyzed by our standard analysis pipeline, including variant calling with GATK, Platypus, VarScan, LoFreq, FreeBayes, SNVer, SAMtools and VarDict and postprocessing of the results. Classification of the reported variants into the categories polymorphisms, mutations and artifacts is performed just like in case of real data. However, as we know about the biological truth in case of the simulated data, accuracy of the classification of all calls is determined.

2 Variant calling

The number of variants reported by the eight variant calling tools in case of the first dataset containing 54 samples and of the second dataset containing 111 samples is summarized in table 4.

Regarding the raw number of calls on target, it is expected that the investigated variant calling tools show considerable differences. Variant calling is always executed with respect to the default recommended options. However, the recommendations concerning e.g. the minimum number of reads supporting the alternate allele differ between the variant calling tools compared. Furthermore, a different set of criteria is evaluated in case of each tool. Regarding GATK, neither a minimum value for the

Table 4. Number of variants on target, after the 3rd filtration, identified mutations, artifacts and polymorphisms, sensitivity (sens), positive predictive value (PPV) and the F_1 score regarding the eight variant calling tools investigated in case of the first and second dataset.

Dataset	Variant caller	Calls on target	3rd filtration	Mutations	Artifacts	Polymorphisms	Sens	PPV	F_1 score
first	GATK	1,650	326	94	55	179	0.83	0.63	0.72
	Platypus	2,122	340	95	67	180	0.84	0.58	0.69
	VarScan	1,341	266	85	7	176	0.75	0.92	0.83
	LoFreq	3743	791	111	503	178	0.98	0.18	0.31
	FreeBayes	35,299	8,211	113	7,918	180	1.00	0.01	0.03
	SNVer	1,860	296	94	24	178	0.83	0.8	0.81
	SAMtools	1,417	285	72	37	178	0.64	0.65	0.65
	VarDict	2,759	292	109	9	176	0.96	0.92	0.94
	Ground truth			113	0	180			
second	GATK	1,559	729	156	209	364	0.69	0.43	0.53
	Platypus	3,095	1385	189	791	405	0.83	0.19	0.31
	VarScan	1,239	499	91	109	299	0.4	0.46	0.43
	LoFreq	150,544	2,141	178	1650	313	0.79	0.1	0.17
	FreeBayes	49,978	35,645	178	35,126	341	0.79	0.01	0.01
	SNVer	7,505	4,735	127	4,389	219	0.56	0.03	0.05
	SAMtools	1,343	518	73	111	334	0.32	0.40	0.36
	VarDict	12,196	4111	205	3,517	389	0.91	0.06	0.10
	Ground truth			226	0	406			

VAF, nor for the coverage can be defined at all, while a minimum VAF, a minimum number of supporting reads for a variant and a minimum depth are defined in case of VarScan.

A comparison of the different variant calling tools on the basis of the calls on target reveals the expected differences. VarScan reports the lowest number of calls in case of both datasets (first set: 1,341; second set: 1,239). FreeBayes and LoFreq, on the contrary, report the highest numbers by far (first set: FreeBayes: 35,299; LoFreq: 3,743; second set: FreeBayes: 49,978; LoFreq: 150,544). The difference in the number of calls reported by LoFreq indicates a considerable difference between the two datasets.

If the number of called variants after the 3rd filtration step is considered, it can be assumed that data is largely free from distorting effects evoked by different internal thresholds of the tools. The applied thresholds of $\#ALT < 20$, $DP < 50$ and $VAF < 1\%$ are stricter than the default ones. However, it can be observed that the overall tendency remains unchanged – VarScan still reports the lowest number of calls (first set: 266; second set: 499), while FreeBayes still reports the highest numbers by far (first set: 8,211; second set: FreeBayes: 35,645). This observation indicates a high sensitivity of FreeBayes in comparison to VarScan. However, as data is unlikely to contain more than 8,000, resp. 35,000 true mutations, FreeBayes is expected to feature a low positive predictive value (PPV).

2.1 Polymorphisms in the real datasets

To test the general performance of the different variant callers, their ability to detect polymorphisms is investigated. Figure 1 sums up the characteristics of polymorphisms in the first- and second dataset in comparison to mutations and artifacts.

A majority of polymorphisms features a VAF close to 1.0 or 0.5. Regarding the influence of the variant on the protein, both SIFT and Provean predict a tolerable effect for a majority of polymorphisms. However, Provean predicts such an effect for almost 100% of the polymorphisms, while SIFT predicts a damaging effect for almost 40% of the polymorphisms in both datasets. Thus, Provean appears to be a more accurate prediction tool.

A vast majority of polymorphisms is present in the databases ESP6500, 1000 Genomes, dbSNP, ExAC and ClinVar (common). No variants feature a "PM flag" in dbSNP, or are present in ClinVar (clinical). However, it can be observed, that roughly 60% of the polymorphisms in both datasets do have at least one Cosmic entry. This observation matches our initial assumption that no database is free from flaws.

Due to their high allelic frequencies, it is expected that all tools succeed in calling the 180 polymorphisms, which are present in the first dataset, as well as the 406 polymorphisms, which are present in the second dataset. However, regarding the first set, only two tools – FreeBayes and Platypus – report all 180 polymorphisms. All the other tools miss one to four polymorphisms (see table 4). Sensitivity with respect to polymorphisms would thus range between 0.98 and 1.0.

Altogether, 168 polymorphisms (93%) are consistently reported by all variant calling tools. The polymorphisms contain 166 SNPs and two indel polymorphisms. 85 out of the 168 polymorphisms (51%) are likely to be homozygous, as their frequency in the alignment data is close to 1.0.

All of the twelve partly missed polymorphisms are SNPs. Three out of twelve (25%) are likely to be homozygous. Ten out of twelve are only missed by one variant caller. These include the three homozygous SNPs. None of the missed SNPs features a low coverage, nor a low mean base quality. Investigation of the corresponding regions in the IGV reveals that they

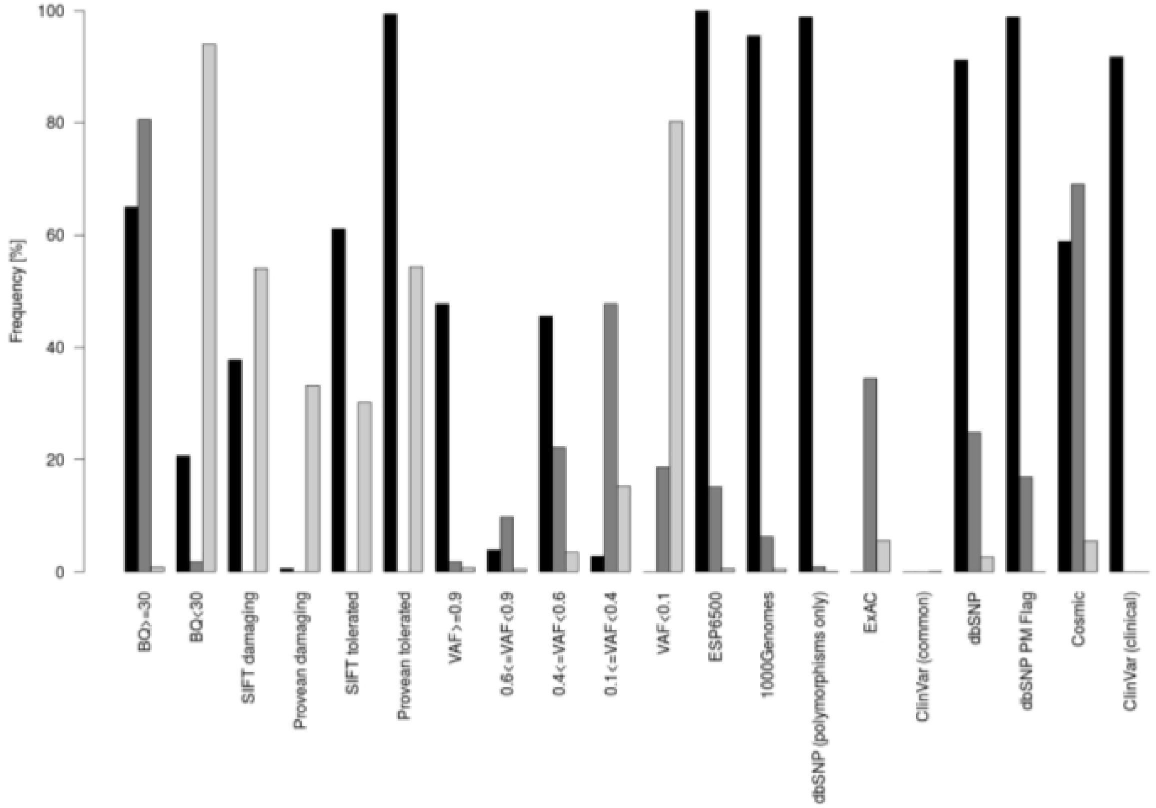

a)

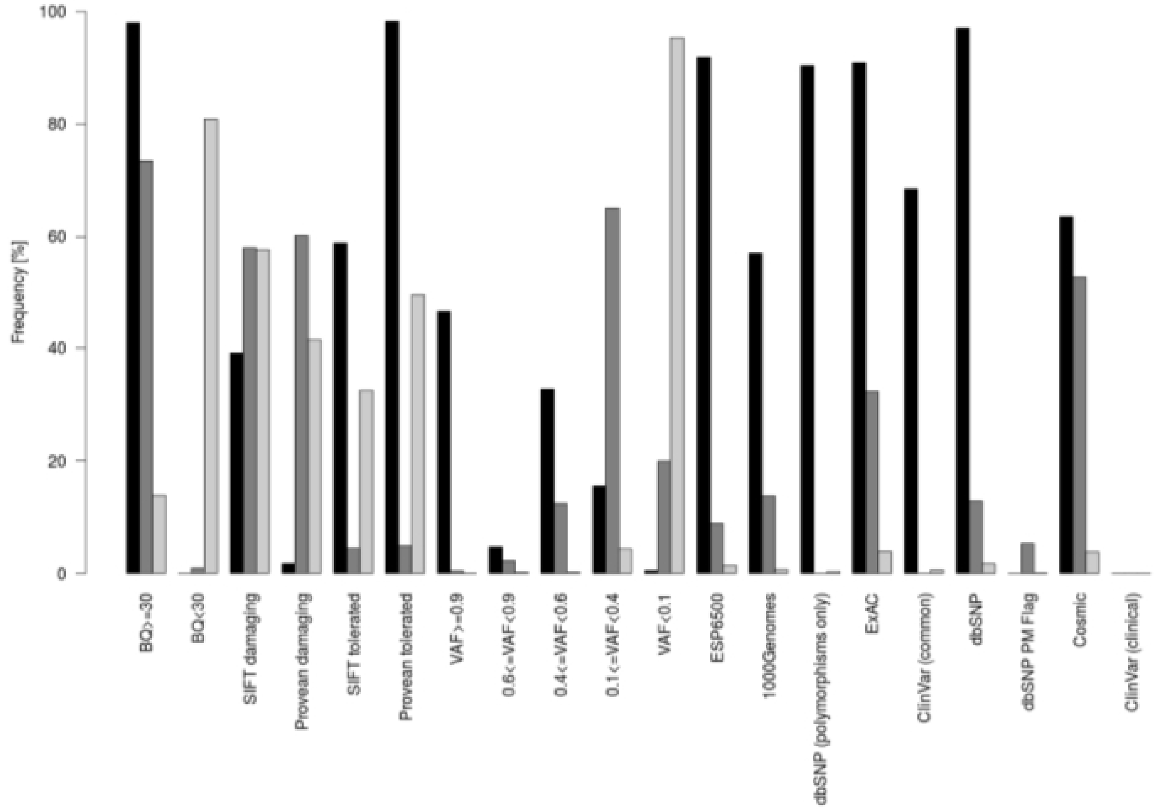

b)

Figure 1. Characteristics of the polymorphisms, mutations and artifacts present in the a) first dataset and b) second dataset.

are covered by different overlapping amplicons. The polymorphisms are always present in all amplicons.

Two of the missed SNPs are missed by two, resp. three variant callers. Different from the other polymorphisms, they feature relatively low VAFs of 0.33, resp. 0.11. However, as we aimed at calling variants with frequencies as low as 1%, both SNPs are covered by more than 100 reads and the mean base qualities of the alternate allele are >34, variant calling should not be a problem.

Regarding the second dataset, variant calling results with respect to polymorphisms are considerably worse. No tool succeeds in calling all polymorphisms. Platypus performs best, missing only one out of 406 polymorphisms. SNVer however only calls 54% of all polymorphisms.

Altogether, only 118 polymorphisms (29%) are consistently reported by all variant calling tools – all of them being SNPs. 43 out of the 118 polymorphisms (36%) are likely to be homozygous due to their VAF.

Eight out of 288 polymorphisms missed by at least one variant calling tool are indel polymorphisms. 124 out of 288 (43%) are likely to be homozygous. It seems remarkable that 126 polymorphisms are missed by exactly one tool, i.e. in 91% of all cases SNVer. Inspection of the missed SNPs does not reveal any reason, why so many clear calls are missed by this variant calling tool.

Out of the remaining 162 polymorphisms, 158 are missed by two to four variant calling tools. These include the eight indel polymorphisms. In all cases, VAF is higher than 20%, coverage is considerably higher than 100 and the mean base qualities of the alternate allele is > 32.

Three SNPs are missed by five variant callers, while one is missed by six. Their VAFs range between 7% and 23%, which is likely to result from unequal amplification of the reads. However, as coverage as well as mean base qualities are high, variant calling should be unproblematic.

2.2 Assessing the negative predictive value

In order to assess the negative predictive value (NPV), we simulate additional 100 samples – 50 on Illumina HiSeq and 50 on Illumina NextSeq. In both cases we choose coverage and error profile of the original data (E1 and C3 in case of HiSeq and E1 and C2 in case of NextSeq). In case of all samples we consider a set of 10 polymorphisms in the analyzed target region that have not been called in any of the analyzed real samples. These polymorphisms serve as controls in order to assess the negative predictive value. The results are summed up in table 5.

Table 5. Number of variants on target, after the 3rd filtration, identified polymorphisms (not called in any of the real samples) and artifacts, sensitivity (sens), positive predictive value (PPV) and the F_1 score regarding the eight variant calling tools investigated in case of the simulated HiSeq- and NextSeq dataset.

Dataset	Variant caller	Calls on target	3rd filtration	Polymorphisms	Artifacts	Sens	PPV	F_1 score
SIM1_E1_C3_NPV	GATK	169	168	168	0	0.99	1.00	0.99
	Platypus	170	169	169	0	0.99	1.00	1.00
	VarScan	165	164	164	0	0.96	1.00	0.98
	LoFreq	164	146	146	0	0.86	1.00	0.92
	FreeBayes	125,288	173	170	3	1.00	0.98	0.99
	SNVer	170	169	169	0	0.99	1.00	1.00
	SAMtools	142	141	141	0	0.83	1.00	0.91
	VarDict	177	173	169	4	0.99	0.98	0.99
Ground truth				170	0			
SIM2_E1_C2_NPV	GATK	184	184	184	0	1.00	1.00	1.00
	Platypus	184	184	184	0	1.00	1.00	1.00
	VarScan	177	177	177	0	0.96	1.00	0.98
	LoFreq	198	177	169	8	0.92	0.95	0.94
	FreeBayes	207	192	184	8	1.00	0.96	0.98
	SNVer	191	186	184	2	1.00	0.99	0.99
	SAMtools	162	162	162	0	0.88	1.00	0.94
	VarDict	184	184	184	0	1.00	1.00	1.00
Ground truth				184	0			

The results clearly show that in case of simulated HiSeq data, FreeBayes features $sens = 1$, while GATK, Platypus, SNVer and VarDict only miss 1-2 simulated polymorphisms. In case of simulated NextSeq data, GATK, Platypus, FreeBayes, SNVer and VarDict even feature $sens = 1$. Despite varying allelic frequencies, there exists no polymorphism in any dataset that was missed by all variant calling tools. The observation that five out of eight tools even feature nearly perfect till perfect sensitivity can be taken as evidence for the NPV also being high in the real datasets – especially when integrating the output of all evaluated tools.

2.3 Influence of varying coverage

The influence of varying coverage on variant calling in the simulated HiSeq dataset is summed up in table S6. The results considering the simulated NextSeq dataset are summed up in table S7.

Table 6. Number of variants on target, after the 3rd filtration, identified mutations, artifacts and polymorphisms, sensitivity (sens), positive predictive value (PPV) and the F_1 score regarding the eight variant calling tools investigated in case of the simulated HiSeq dataset.

Dataset	Variant caller	Calls on target	3rd filtration	Mutations	Artifacts	Polymorphisms	Sens	PPV	F_1 score
SIM1_E1_C1	GATK	260	250	91	9	150	0.54	0.91	0.67
	Platypus	268	258	90	21	147	0.53	0.81	0.64
	VarScan	220	210	73	0	137	0.43	1.00	0.60
	LoFreq	343	297	115	65	117	0.68	0.64	0.66
	FreeBayes	42,232	2,722	124	2,481	117	0.73	0.05	0.09
	SNVer	310	294	123	21	150	0.72	0.85	0.78
	SAMtools	207	197	80	0	117	0.47	1.00	0.64
	VarDict	344	330	124	57	149	0.73	0.69	0.71
SIM1_E1_C2	GATK	271	261	94	11	156	0.55	0.90	0.68
	Platypus	281	271	94	21	156	0.55	0.82	0.66
	VarScan	224	214	71	0	143	0.42	1.00	0.59
	LoFreq	333	295	116	57	122	0.68	0.67	0.68
	FreeBayes	49,099	3,747	126	3,499	122	0.74	0.03	0.07
	SNVer	317	303	124	23	156	0.73	0.84	0.78
	SAMtools	214	204	82	0	122	0.48	1.00	0.65
	VarDict	359	347	125	66	156	0.74	0.65	0.69
SIM1_E1_C3	GATK	283	273	100	11	162	0.59	0.90	0.71
	Platypus	294	284	101	21	162	0.59	0.83	0.69
	VarScan	237	227	77	0	150	0.45	1.00	0.62
	LoFreq	299	279	122	29	128	0.72	0.81	0.76
	FreeBayes	125,024	30,113	131	29,854	128	0.77	0.004	0.01
	SNVer	327	314	130	22	162	0.76	0.86	0.81
	SAMtools	223	213	85	0	128	0.50	1.00	0.67
	VarDict	363	349	131	56	162	0.77	0.70	0.73
SIM1_E1_C4	GATK	284	274	98	11	165	0.58	0.90	0.70
	Platypus	295	285	99	21	165	0.58	0.83	0.68
	VarScan	241	231	77	0	154	0.45	1.00	0.62
	LoFreq	285	267	120	16	131	0.71	0.88	0.78
	FreeBayes	366,885	762	126	505	131	0.74	0.20	0.31
	SNVer	320	309	125	19	165	0.74	0.87	0.80
	SAMtools	222	212	81	0	131	0.48	1.00	0.65
	VarDict	360	347	129	53	165	0.76	0.71	0.73
SIM1_E1_C5	GATK	288	274	99	11	168	0.58	0.90	0.71
	Platypus	302	285	100	24	168	0.59	0.81	0.68
	VarScan	240	231	77	0	153	0.45	1.00	0.62
	LoFreq	272	267	122	4	134	0.72	0.97	0.82
	FreeBayes	737,024	311	112	65	134	0.66	0.63	0.65
	SNVer	317	309	124	14	168	0.73	0.90	0.81
	SAMtools	229	212	85	0	134	0.50	1.00	0.67
	VarDict	403	347	131	84	168	0.77	0.61	0.68
Ground truth				170	0	170			

As polymorphisms in the simulated datasets are designed to be present with frequencies close to 0.50 or 1.00, varying coverage is not expected to harm the calling of polymorphisms.

Different from our expectations, a general decrease in sensitivity concerning polymorphisms can be observed as coverage increases. The decrease is barely visible in case of the simulated NextSeq data and might therefore be due to random effects. In contrast to this, it is clearly visible in case of the simulated HiSeq data. All tools report less polymorphisms at 6,000x coverage compared to 500x coverage.

If the performance of the different tools is considered in general and not in relation to varying coverage, GATK, Platypus, SNVer and VarDict do all perform best on the two different sets of simulated data. Regarding the simulated NextSeq data, the tools partly succeed in calling all polymorphisms. Regarding the simulated HiSeq data, no tool manages to call all polymorphisms. However, the four tools mentioned miss – in case of the best scenario – only two out of 170 polymorphisms.

Table 7. Number of variants on target, after the 3rd filtration, identified mutations, artifacts and polymorphisms, sensitivity (sens), positive predictive value (PPV) and the F_1 score regarding the eight variant calling tools investigated in case of the simulated NextSeq dataset.

Dataset	Variant caller	Calls on target	3rd filtration	Mutations	Artifacts	Polymorphisms	Sens	PPV	F_1 score
SIM2_E1_C1	GATK	313	298	116	0	182	0.63	1.00	0.77
	Platypus	328	313	131	0	182	0.71	1.00	0.83
	VarScan	249	234	77	0	157	0.42	1.00	0.59
	LoFreq	451	415	127	147	141	0.69	0.46	0.55
	FreeBayes	455	407	164	61	182	0.89	0.73	0.80
	SNVer	431	398	151	65	182	0.82	0.70	0.76
	SAMtools	233	218	77	0	141	0.42	1.00	0.59
	VarDict	372	357	167	8	182	0.91	0.95	0.93
SIM2_E1_C2	GATK	311	296	113	0	183	0.61	1.00	0.76
	Platypus	330	315	131	1	183	0.71	0.99	0.83
	VarScan	261	246	79	0	167	0.43	1.00	0.60
	LoFreq	437	402	127	133	142	0.69	0.49	0.57
	FreeBayes	457	409	164	62	183	0.89	0.73	0.80
	SNVer	427	394	151	60	183	0.82	0.72	0.76
	SAMtools	235	220	78	0	142	0.42	1.00	0.60
	VarDict	375	360	167	10	183	0.91	0.94	0.93
SIM2_E1_C3	GATK	314	299	115	0	184	0.63	1.00	0.77
	Platypus	336	321	131	6	184	0.71	0.96	0.82
	VarScan	258	243	78	0	165	0.42	1.00	0.60
	LoFreq	396	363	127	93	143	0.69	0.58	0.63
	FreeBayes	473	413	164	65	184	0.89	0.72	0.79
	SNVer	419	386	151	51	184	0.82	0.75	0.78
	SAMtools	238	223	80	0	143	0.43	1.00	0.61
	VarDict	372	357	167	6	184	0.91	0.97	0.94
SIM2_E1_C4	GATK	314	299	115	0	184	0.63	1.00	0.77
	Platypus	335	319	130	5	184	0.71	0.96	0.82
	VarScan	257	242	78	0	164	0.42	1.00	0.60
	LoFreq	369	336	127	66	143	0.69	0.66	0.67
	FreeBayes	509	413	163	66	184	0.89	0.71	0.79
	SNVer	398	365	151	30	184	0.82	0.83	0.83
	SAMtools	237	222	79	0	143	0.43	1.00	0.60
	VarDict	376	359	167	8	184	0.91	0.95	0.93
SIM2_E1_C5	GATK	316	301	117	0	184	0.64	1.00	0.78
	Platypus	333	318	131	3	184	0.71	0.98	0.82
	VarScan	262	247	79	0	168	0.43	1.00	0.60
	LoFreq	331	300	127	30	143	0.69	0.81	0.74
	FreeBayes	621	447	163	100	184	0.89	0.62	0.73
	SNVer	373	346	144	18	184	0.78	0.89	0.83
	SAMtools	236	221	78	0	143	0.42	1.00	0.60
	VarDict	385	365	167	14	184	0.91	0.92	0.92
Ground truth				184	0	184			

2.4 Influence of varying background noise

The influence of varying background noise on variant calling in the simulated HiSeq dataset is summed up in table S8. The results considering the simulated NextSeq dataset are summed up in table S9.

Table 8. Number of variants on target, after the 3rd filtration, identified mutations, artifacts and polymorphisms, sensitivity (sens), positive predictive value (PPV) and the F_1 score at different levels of background noise regarding the eight variant calling tools investigated in case of the simulated HiSeq dataset.

Dataset	Variant caller	Calls on target	3rd filtration	Mutations	Artifacts	Polymorphisms	Sens	PPV	F_1 score
SIM1_E2_C3	GATK	282	272	98	4	170	0.58	0.96	0.72
	Platypus	295	285	100	15	170	0.59	0.87	0.70
	VarScan	232	222	76	0	146	0.45	1.00	0.62
	LoFreq	330	320	122	62	136	0.72	0.66	0.69
	FreeBayes	55223	11640	131	11340	169	0.77	0.01	0.02
	SNVer	356	336	130	36	170	0.76	0.78	0.77
	SAMtools	224	214	78	0	136	0.46	1.00	0.63
	VarDict	732	335	130	35	170	0.76	0.79	0.78
SIM1_E3_C3	GATK	287	276	100	8	168	0.59	0.93	0.72
	Platypus	299	288	101	19	168	0.59	0.84	0.70
	VarScan	238	227	77	0	150	0.45	1.00	0.62
	LoFreq	402	339	122	83	134	0.72	0.60	0.65
	FreeBayes	5003	389	131	89	169	0.77	0.60	0.67
	SNVer	359	335	131	36	168	0.77	0.78	0.78
	SAMtools	229	218	84	0	134	0.49	1.00	0.66
	VarDict	710	335	131	36	168	0.77	0.78	0.78
Ground truth				170	0	170			

Table 9. Number of variants on target, after the 3rd filtration, identified mutations, artifacts and polymorphisms, sensitivity (sens), positive predictive value (PPV) and the F_1 score at different levels of background noise regarding the eight variant calling tools investigated in case of the simulated NextSeq dataset.

Dataset	Variant caller	Calls on target	3rd filtration	Mutations	Artifacts	Polymorphisms	Sens	PPV	F_1 score
SIM2_E2_C2	GATK	312	297	113	0	184	0.61	1.00	0.76
	Platypus	330	315	131	0	184	0.71	1.00	0.83
	VarScan	256	241	79	0	162	0.43	1.00	0.60
	LoFreq	395	362	127	92	143	0.69	0.58	0.63
	FreeBayes	516	418	164	70	184	0.89	0.70	0.78
	SNVer	410	377	151	42	184	0.82	0.78	0.80
	SAMtools	230	215	72	0	143	0.39	1.00	0.56
	VarDict	379	364	167	13	184	0.91	0.93	0.92
SIM2_E3_C2	GATK	315	299	116	0	183	0.63	1.00	0.77
	Platypus	339	323	130	9	184	0.71	0.94	0.80
	VarScan	260	244	78	0	166	0.42	1.00	0.60
	LoFreq	461	421	127	152	142	0.69	0.46	0.55
	FreeBayes	450	406	163	59	184	0.89	0.73	0.80
	SNVer	400	366	151	32	183	0.82	0.83	0.82
	SAMtools	240	224	82	0	142	0.45	1.00	0.62
	VarDict	367	351	167	0	184	0.91	1.00	0.95
Ground truth				184	0	184			

Different from actual mutations, polymorphisms were designed to be present in the simulated datasets with allelic frequencies close to 0.50 or 1.00. Similar to varying coverage, varying background noise is thus not expected to harm the calling of polymorphisms.

The results we observe measure up to our expectations. The results concerning E2 and E3 hardly differ. As no clear tendency is visible, it seems apt to assume, that all observed differences are due to random variation in the simulated datasets.

3 Figures and tables

Table 10. Variant calling tools excluded from consideration.

Tool	Exclusion criteria	Reference
AbsoluteVar	not available	[8]
Altools	using VarScan	[3]
ASAP	download link not working	[34]
Atlas2	Ruby 2.3.0 incompatible	[4]
bambino	reference not accepted,<br>no calls without reference	[12]
bcbio-nextgen	combination of various variant callers	bcbio-nextgen.readthedocs.io
CAKE	combination of Bambino, CaVEMan,<br>SAMtools and VarScan2	[28]
CaVEMan	matched samples required	[33]
CRISP	matched samples required	[2]
DeepSNV	matched samples required	[16]
Dindel	indel calling only	[1]
EBCall	matched samples required	[31]
GATK UnifiedGenotyper	deprecated	[9]
gkno	using FreeBayes	gkno.me/
inGAP	inappropriate output format	[26]
Isaac variant caller	calls lacking alternate allele<br>or not passing internal filters	[20]
JointSNVMix1 and 2	matched samples required	[29]
MutationSeq	matched samples required	[10]
MuTect2	matched samples required	[7]
NGSEP	pipeline not working	[11]
QQ-SNV	SAS script only	[36]
QuadGT	four related genomes required	www.iro.umontreal.ca/ csuros/quadgt/
Scalpel 0.5.2	indel calling only	[14]
ScanIndel	indel calling only	[37]
Seurat 2.5	matched samples required	[6]
Shimmer	matched samples required	[19]
SNVMix 0.11.8	download link not working	[17]
SOAPsnv 2.0 and SOAPindel 2.1	pipeline not working	[23]
SolSNP 1.11	SNV calling only	[32]
SomaticSniper 1.0.5.0	matched samples required	[21]
SpeedSeq	using FreeBayes	[5]
Splinter	matched samples required	[35]
Strelka 1.0.14	matched samples required	[30]
thunder	building script failed	[24]
Virmid 1.1.1	matched samples required	[25]

Table 11. Overview of the variant callers investigated and the commands.

Tool	Command
GATK	Pre-processing:<br>`java -jar GenomeAnalysisTK.jar -T BaseRecalibrator -I sampleX.bam -R Ref_GRCh37.67.fasta`<br>`--maximum_cycle_value 1500 --covariate ContextCovariate --covariate CycleCovariate`<br>`--covariate QualityScoreCovariate --covariate ReadGroupCovariate -knownSites dbSNP.vcf`<br>`-knownSites MillsAnd1000GGoldStandard.indels.vcf -knownSites 1000GPhase1.indels.vcf`<br>`-o RecalData.csv`<br>`java -jar GenomeAnalysisTK.jar -T PrintReads -I sampleX.bam -R Ref_GRCh37.67.fasta`<br>`-BQSR RecalData.csv -o sampleX_r.bam`<br>`java -jar BuildBamIndex.jar VALIDATION_STRINGENCY="LENIENT" INPUT=sampleX_r.bam`<br>`OUTPUT=sampleX_r.bai`<br>Variant Calling:<br>`java -jar GenomeAnalysisTK.jar -T HaplotypeCaller -R Ref_GRCh37.67.fasta -stand_call_conf 30.0`<br>`-stand_emit_conf 10.0 --dbsnp dbSNP.polymorphisms.vcf -L target.bed -I sampleX_r.bam`<br>`-o sampleX.vcf`
Platypus	`python Platypus.py callVariants --bamFiles=sampleX.bam --refFile Ref_GRCh37.67.fasta`<br>`--output=sampleX.vcf --filterDuplicates=0 --minflank=0`
VarScan	Pre-processing:<br>`samtools mpileup -f Ref_GRCh37.67.fasta samplex.bam > sampleX.bcf`<br>Variant calling:<br>`java -jar VarScan.v2.3.9.jar mpileup2snp sampleX.bcf > sampleX.snvs.txt`<br>`java -jar VarScan.v2.3.9.jar mpileup2indels sampleX.bcf > sampleX.indels.txt`
LoFreq	Pre-processing:<br>`lofreq indelqual --dindel -f Ref_GRCh37.67.fasta -o sampleX_q.bam sampleX.bam`<br>`samtools index -b sampleX_q.bam > sampleX_q.bai`<br>Variant calling:<br>`lofreq call --call-indels -f Ref_GRCh37.67.fasta -o sampleX.vcf sampleX_q.bam -s`<br>`-S dbSNP.polymorphisms.vcf.gz`
FreeBayes	`freebayes -F 0.01 -f Ref_GRCh37.67.fasta sampleX.bam > sampleX.vcf`
SNVer	`java -jar SNVerIndividual.jar -i sampleX.bam -r Ref_GRCh37.67.fasta -b 0.01 -o sampleX.vcf`
SAMtools	Pre-processing:<br>`samtools mpileup -q 1 -g -u -o sampleX.bcf -f Ref_GRCh37.67.fasta sampleX.bam`<br>Variant calling:<br>`bcftools call -vmO v -o sampleX.vcf sampleX.bcf`
VarDict	`AF_THR="0.01"`<br>`vardict -C -G Ref_GRCh37.67.fasta -f $AF_THR -N sampleX -b sampleX.bam -h -c 1 -S 2`<br>`-E 3 -g 4 target.bed > sampleX.txt`

Table 12. List of the genes, exons and their ENSEMBL ([15]) transcript IDs that were targeted by Illumina HiSeq (first dataset) and Illumina NextSeq (second dataset).

Gene	Exons	TranscriptID
ASXL1	E13	ENST00000375687
CBL	E8, E9	ENST00000264033
CEBPA	E1	ENST00000498907
DNMT3A	E2-E23	ENST00000264709
ETV6	E1-E8	ENST00000396373
EZH2	E2-E20	ENST00000320356
FLT3	E20	ENST00000241453
IDH1	E4	ENST00000345146
IDH2	E4	ENST00000330062
JAK2	E12, E14	ENST00000381652
KRAS	E2, E3	ENST00000256078
NRAS	E2, E3	ENST00000369535
RUNX1	E4-E9	ENST00000437180
SF3B1	E13-E16	ENST00000335508
SRSF2	E1	ENST00000392485
TET2	E3-E11	ENST00000380013
TP53	E2-E11	ENST00000269305
U2AF1	E2, E6	ENST00000291552
ZRSR2	E1-E11	ENST00000307771

Table 13. Output files and information they contain in case of the eight variant calling tools investigated.

Variant caller	Output file	Information
GATK	1 vcf file	CHROM, POS, ID, REF, ALT, QUAL, FILTER (LowQual), INFO (AC, AF, AN, BaseQRankSum, ClippingRankSum, DB, DP, DS, FS, HaplotypeScore, InbreedingCoeff, MLEAC, MLEAF, MQ, MQ0, MQRankSum, QD, ReadPosRankSum, SOR), FORMAT (AD, DP, GQ, GT, PL)
Platypus	1 vcf file	CHROM, POS, ID, REF, ALT, QUAL, FILTER (GOF, badReads, alleleBias, hp10, Q20, HapScore, MQ, strandBias, SC, QualDepth, REFCALL, QD), INFO (FR, MMLQ, TCR, HP, WE, Source, FS, WS, PP, TR, NF, TCF, NR, TC, END, MGOF, SbPval, START ReadPosRankSum, MQ, QD, SC, BRF, HapScore, Size), FORMAT (GT, GQ, GOF, NR, GL, NV)
VarScan	1 txt file regarding SNVs	Chrom, Position, Ref, Var, Cons:Cov:Reads1:Reads2:Freq: P-value, StrandFilter:R1+:R1-:R2+:R2-:pval, SamplesRef SamplesHet, SamplesHom, SamplesNC, Cons:Cov:Reads1: Reads2:Freq:P-value
	1 txt file regarding indels	Chrom, Position, Ref, Var, Cons:Cov:Reads1:Reads2:Freq: P-value, StrandFilter:R1+:R1-:R2+:R2-:pval, SamplesRef SamplesHet, SamplesHom, SamplesNC, Cons:Cov:Reads1: Reads2:Freq:P-value
LoFreq	1 vcf file	CHROM, POS, ID, REF, ALT, QUAL, FILTER (min_dp_10, sb_fdr, min_snvqual_76, min_indelqual_61), INFO (DP, AF, SB, DP4, INDEL, CONSVAR, HRUN)
FreeBayes	1 vcf file	CHROM, POS, ID, REF, ALT, QUAL, FILTER, INFO (NS, DP, DPB, AC, AN, AF, RO, AO, PRO, PAO, QR, QA, PQR, PQA, SRF, SRR, SAF, SAR, SRP, SAP, AB, AB, ABP, RUN, RPP, RPPR, RPL, RPR, EPP, EPPR, DPRA, ODDS, GTI, TYPE, CIGAR, NUMALT, MEANALT, LEN, MQM, PAIRED, PAIREDR, MIN, END, technology.ILLUMINA), FORMAT (GT, GQ, GL, DP, DPR, RO, QR, AO, QA, MIN)
SNVer	1 raw vcf file regarding SNVs	CHROM, POS, ID, REF, ALT, QUAL, FILTER, INFO (DP, AC, FS, SP, PV), FORMAT (AC1, AC2, RC1, RC2, GT, PL)
	1 filtered vcf file regarding SNVs	CHROM, POS, ID, REF, ALT, QUAL, FILTER, INFO (DP, AC, FS, SP, PV), FORMAT (AC1, AC2, RC1, RC2, GT, PL)
	1 raw vcf file regarding indels	CHROM, POS, ID, REF, ALT, QUAL, FILTER, INFO (DP, AC, PV), FORMAT (GT, PL)
	1 filtered vcf file regarding indels	CHROM, POS, ID, REF, ALT, QUAL, FILTER, INFO (DP, AC, PV), FORMAT (GT, PL)
SAMtools	1 vcf file	CHROM, POS, ID, REF, ALT, QUAL, FILTER, INFO (INDEL, IDV, IMF, DP, VDB, RPB, MQB, BQB, MQSB, SGB, MQ0F, ICB, HOB, AC, AN, DP4, MQ), FORMAT (PL, GT)
VarDict	1 txt file	Sample, Gene, Chr, Start, End, Ref, Alt, Depth, AltDepth, RefFwdReads, RefRevReads, AltFwdReads, AltRevReads, Genotype, AF, Bias, PMean, PStd, QMean, QStd, 5pFlankSeq, 3pFlankSeq

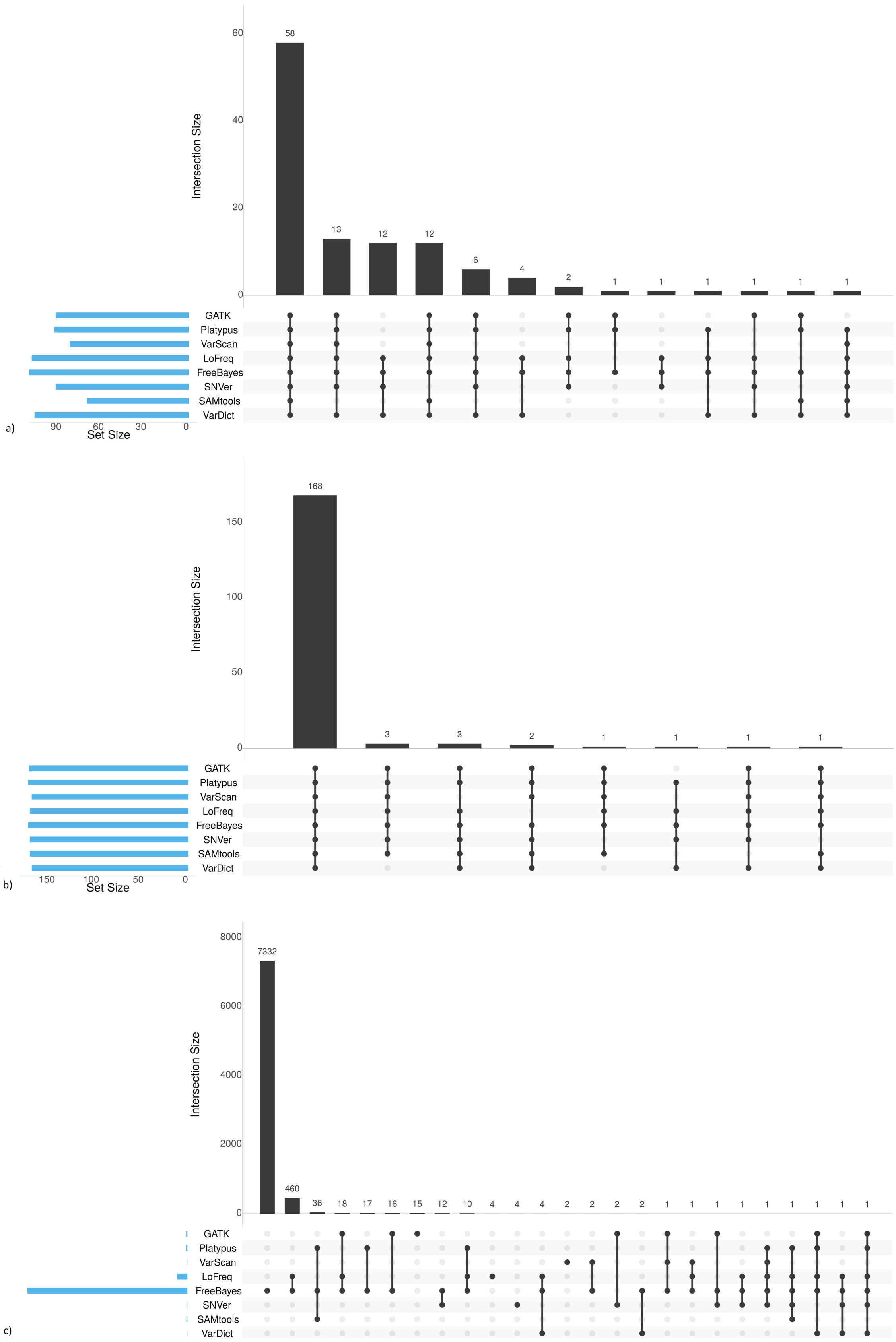

Figure 2. Upset plots considering the degree of overlap between the detected a) mutations, b) polymorphisms, c) artifacts by the different tools in the HiSeq dataset. Black bars show the number of variant calls per category, blue bars show the number of variant calls per caller. E.g. 12 mutations are detected by LoFreq, FreeBayes, SNVer and VarDict, but not by any other tool.

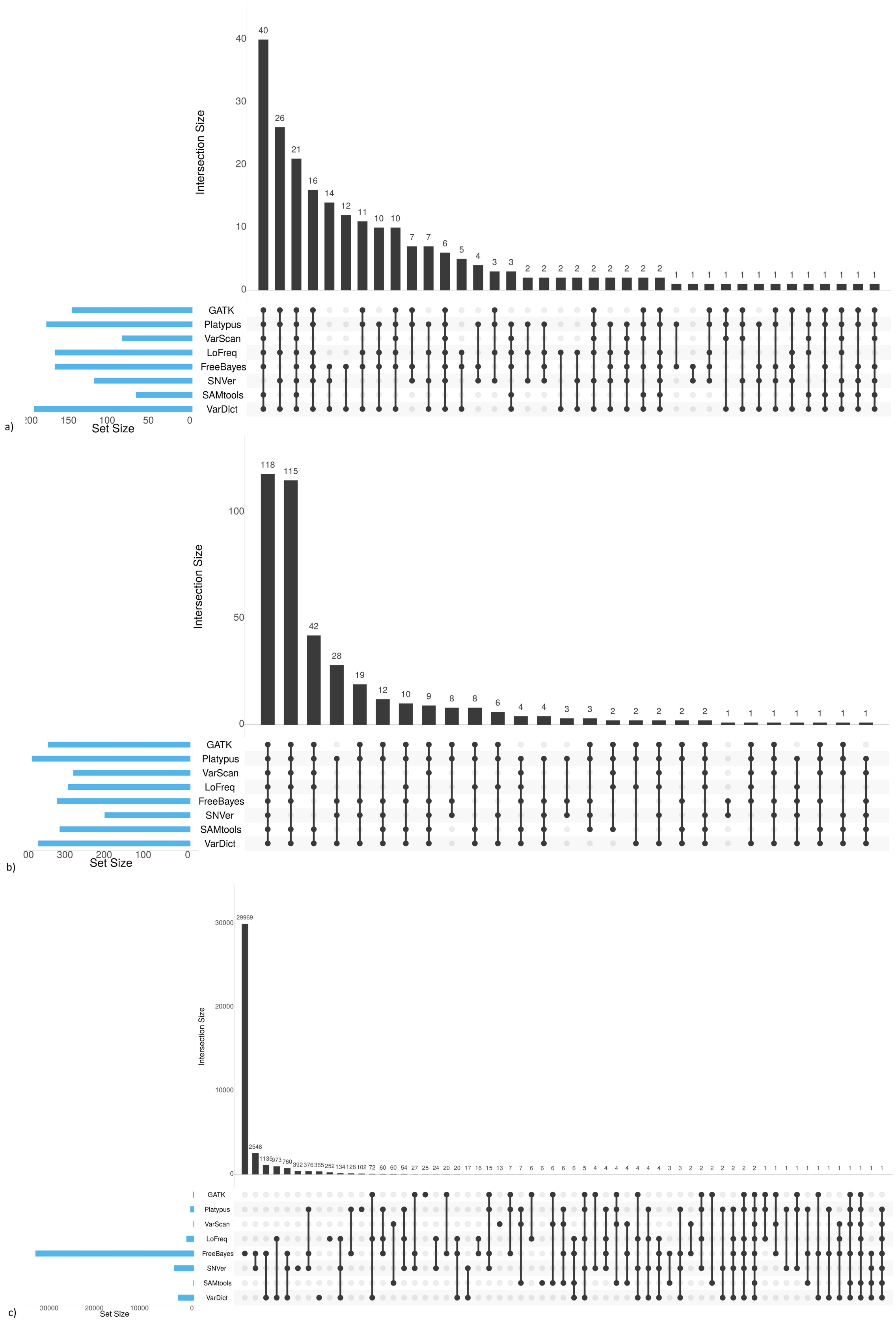

Figure 3. Upset plots considering the degree of overlap between the detected a) mutations, b) polymorphisms, c) artifacts by the different tools in the NextSeq dataset. Black bars show the number of variant calls per category, blue bars show the number of variant calls per caller.

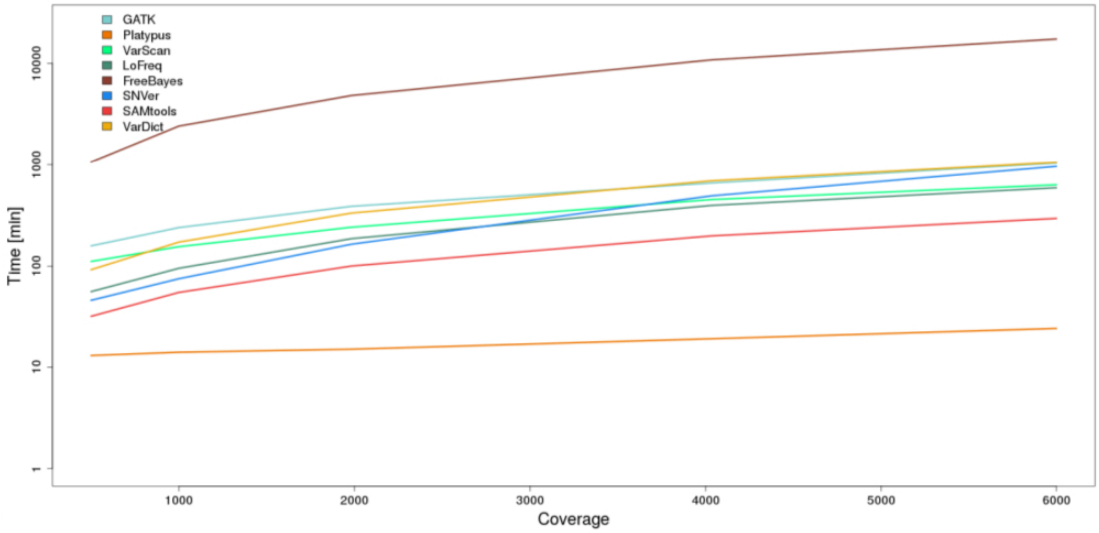

a)

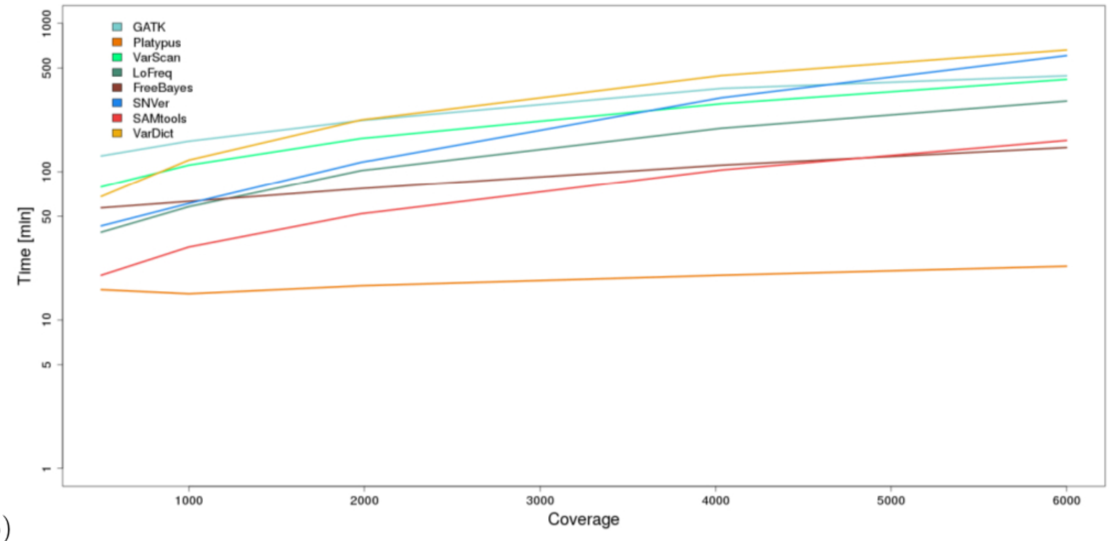

b)

Figure 4. Run time (in minutes) of the variant calling process regarding GATK, Platypus, VarScan, LoFreq, FreeBayes, SNVer, SAMtools and VarDict in the context of increasing coverage: a) simulated data SIM1 (HiSeq), b) simulated data SIM2 (NextSeq).

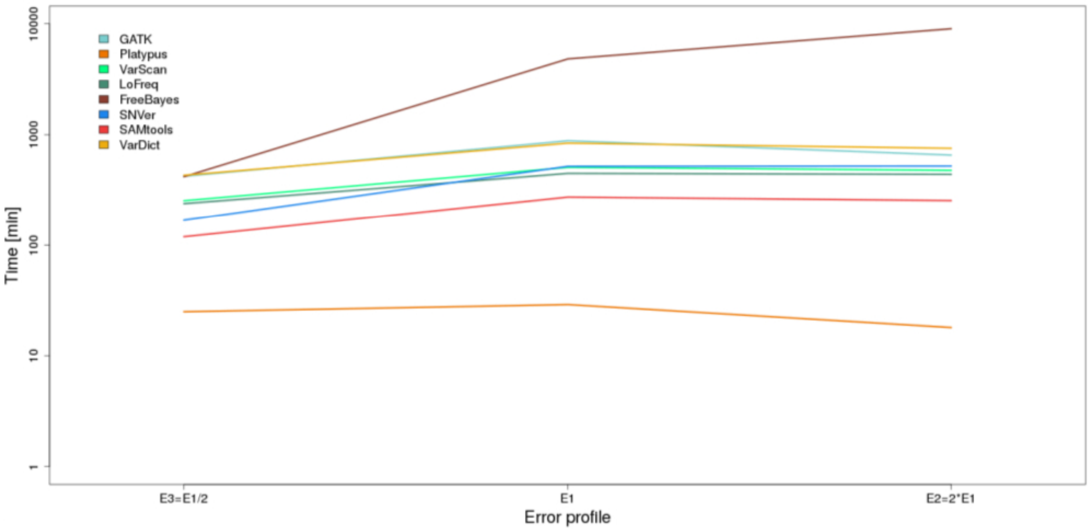

a)

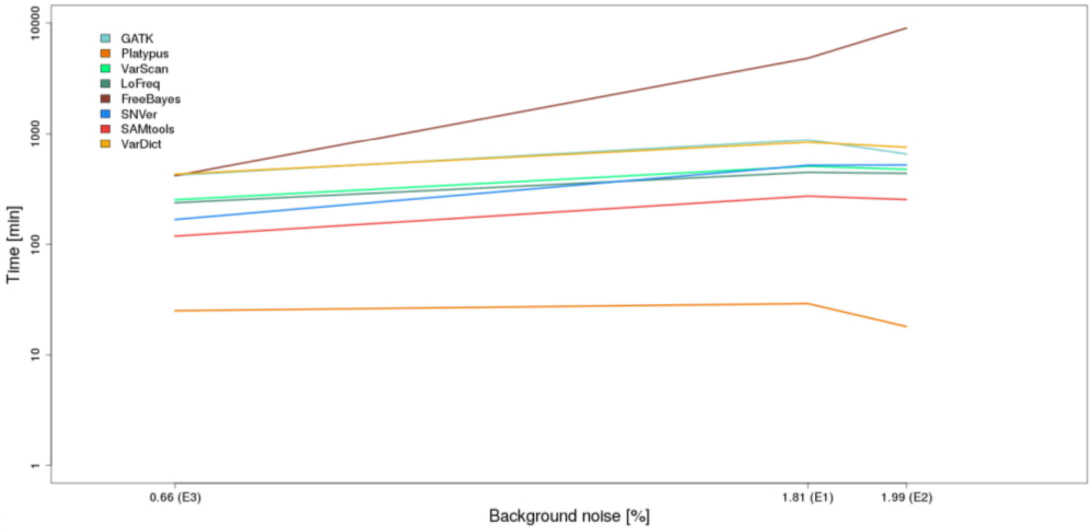

b)

Figure 5. Run time (in minutes) of the variant calling process regarding GATK, Platypus, VarScan, LoFreq, FreeBayes, SNVer, SAMtools and VarDict in the context of varying background noise in simulated data SIM1 (HiSeq): a) run time in relation to simulated error profile, b) run time in relation to determined background noise.

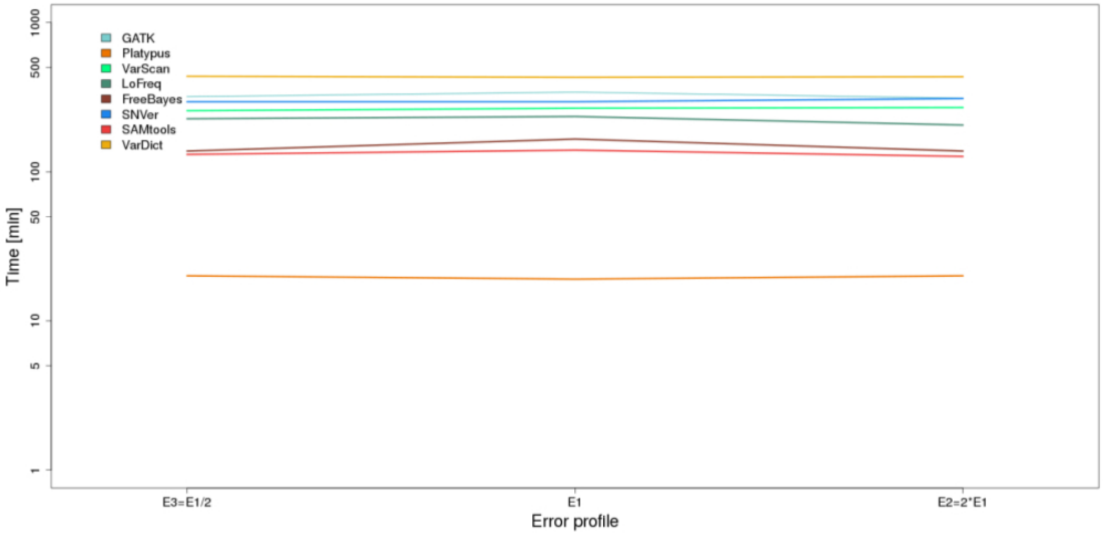

a)

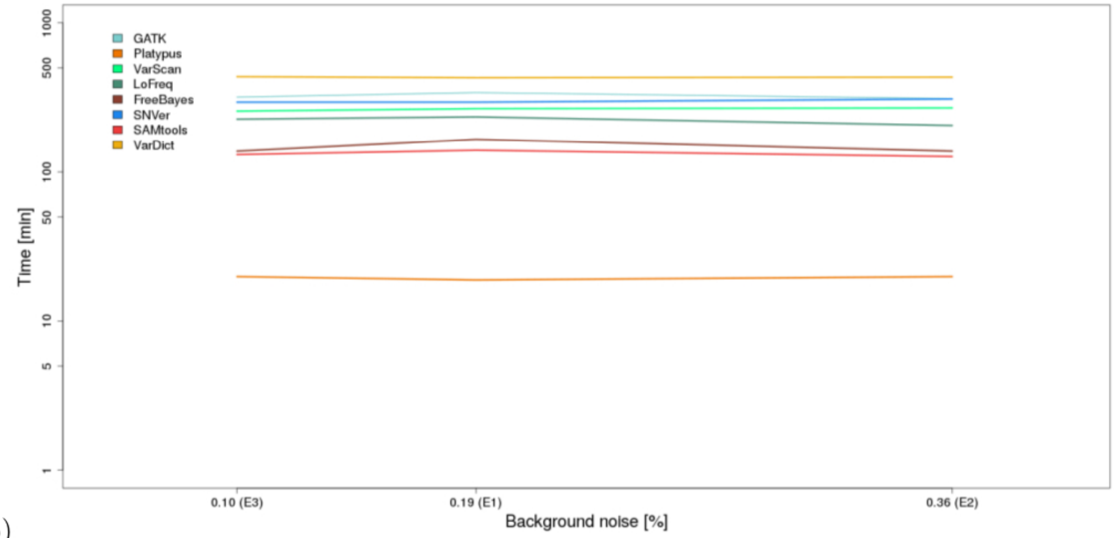

b)

Figure 6. Run time (in minutes) of the variant calling process regarding GATK, Platypus, VarScan, LoFreq, FreeBayes, SNVer, SAMtools and VarDict in the context of varying background noise in simulated data SIM2 (NextSeq): a) run time in relation to simulated error profile, b) run time in relation to determined background noise.

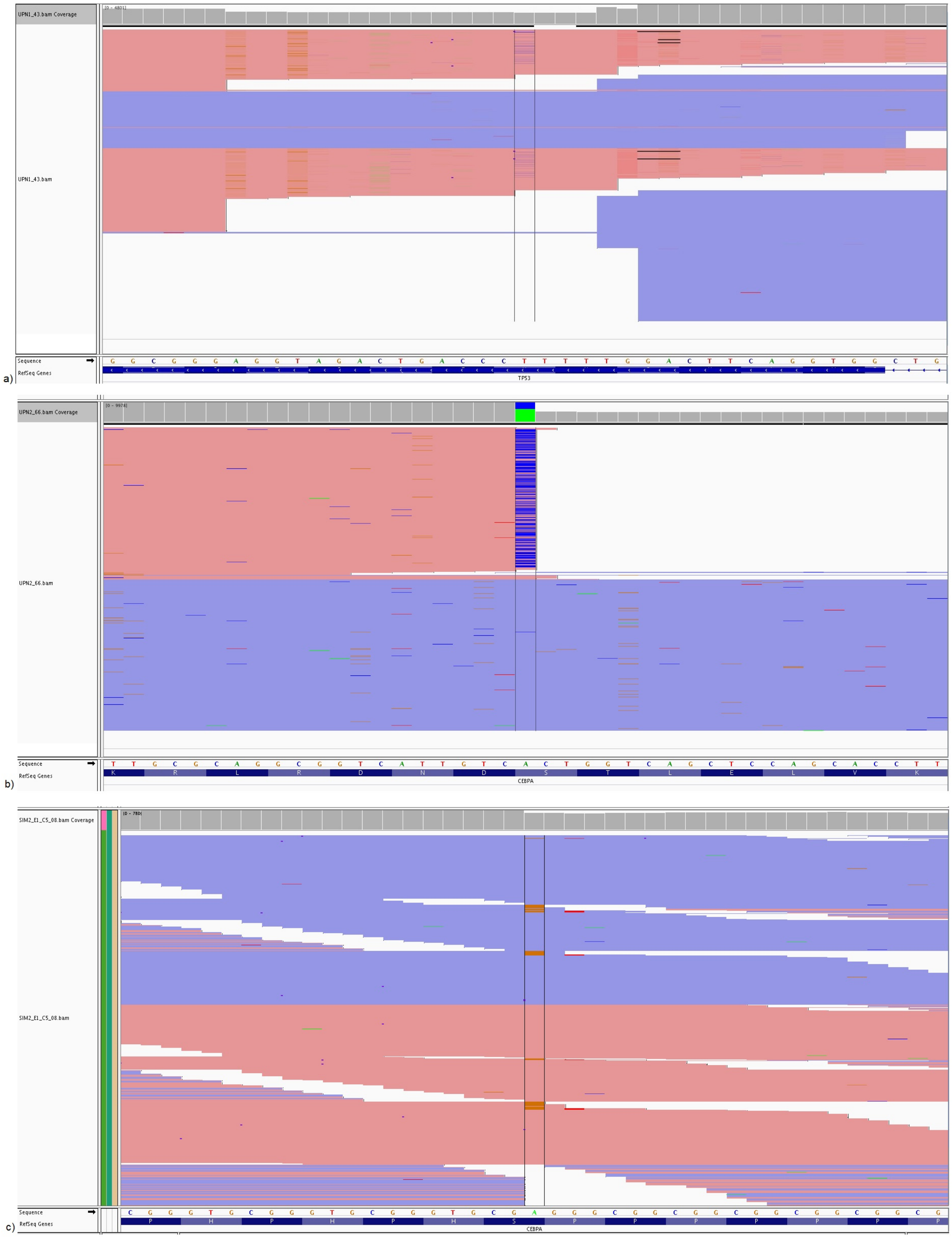

Figure 7. Exemplary artifacts: a) in the real HiSeq data (artifact can be identified by strand bias, low base quality and bad alignment of the forward reads), b) in the real NextSeq data (artifact can be identified by strand bias and position in the forward reads), c) in the simulated NextSeq data.

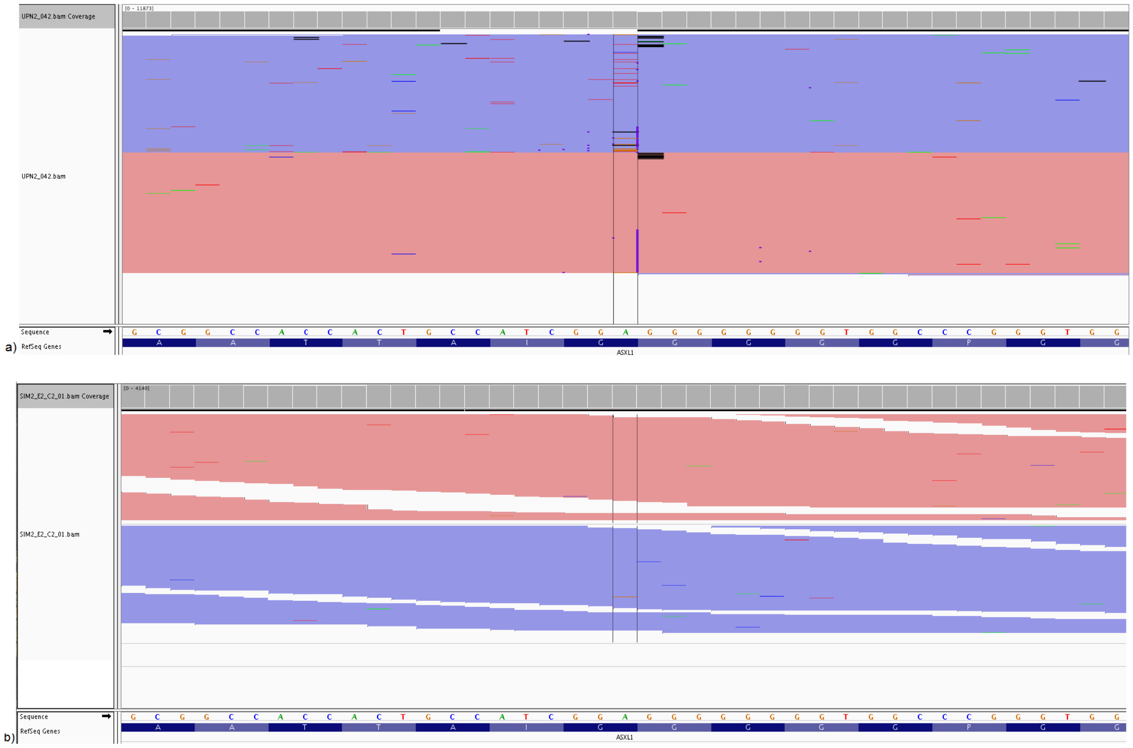

Figure 8. Complex region involving a homopolymeric stretch of eight G's on ASXL1: a) real NextSeq data; although no mutation is present, correct determination of the number of G's is difficult, several reads feature a deletion of one G, an insertion of one G or a mismatching base at the proceeding A, b) simulated NextSeq data (doubled error profile of the original data used for simulation); no increase in sequencing errors can be observed in relation to the complex sequencing context.

References

1. Albers,C.A., Lunter,G., MacArthur,D.G., McVean,G., Ouwehand,W.H., Durbin,R. (2010) Dindel: Accurate indel calls from short-read data, *Genome Res*, **27**, doi:10.1101/gr.112326.110.

2. Bansal,V. (2016) A statistical method for the detection of variants from next-generation resequencing of DNA pools, *Bioinformatics*, **26**, i318-i324, doi:10.1093/bioinformatics/btq214.

3. Camiolo,S., Sablok,G., Porceddu,A. (2016) Altools: a user friendly NGS data analyser, *Biol Direct*, **11**, 8, doi:10.1186/s13062-016-0110-0.

4. Challis,D., Yu,J., Evani,U.S., Jackson,A.R., Paithankar,S., Coarfa,C., Milosavljevic,A., Gibbs,R.A., Yu,F. (2012) An integrative variant analysis suite for whole exome next-generation sequencing data, *BMC Bioinformatics*, **13**, 8, doi:10.1186/1471-2105-13-8.

5. Chiang,C., Layer,R.M., Faust,G.G., Lindberg,M.R., Rose,D.B., Garrison,E.P., Marth,G.T., Quinlan,A.R., Hall,I.M. (2015) SpeedSeq: ultra-fast personal genome analysis and interpretation, *Nat Methods*, **12**, 966-968, doi:10.1038/nmeth.3505.

6. Christoforides,A., Carpenten,J.D., Weiss,G.J., Demeure,M.J., Von Hoff,D.D., Craig,D.W. (2013) Identification of somatic mutations in cancer through Bayesian-based analysis of sequenced genome pairs, *BMC Genomics*, **14**, 302, doi:10.1186/1471-2164-14-302.

7. Cibulskis,K., Lawrence,M.S., Carter,S.L., Sivachenko,A., Jaffee,D., Sougnez,C., Gabriel,S., Meyerson,M., Lander,E.S., Getz,G. (2013) Sensitive detection of somatic point mutations in impure and heterogeneous cancer samples, *Nat Biotechnol*, **31**, 213-219, doi:10.1038/nbt.2514.

8. Daber,R., Sukhadia,S., Morissette,J.J. (2013) Understanding the limitations of next generation sequencing informatics, an approach to clinical pipline validation using artificial data sets, *Cancer Genet*, **206**, 441-8, doi:10.1016/j.cancergen.2013.11.005.

9. DePristo,M., Banks,E., Poplin,R., Garimella,K., Maguire,J., Hartl,C., Philippakis,A.,del Angel,G., Rivas,M.A., Hanna,M., McKenna,A., Fennell,T., Kernytsky,A., Sivachenko,A., Cibulskis,K., Gabriel,S., Altshuler,D., Daly,M. (2011) A framework for variation discovery and genotyping using next-generation DNA sequencing data, *Nat Genet*, **43**, 491-498.

10. Ding,J., Bashashati,A., Roth,A., Oloumi,A., Tse,K., Zeng,T., Haffari,G., Hirst,M., Marra,M.A., Condon,A., Aparicio,S., Shah,S.P. (2012) Feature-based classifiers for somatic mutation detection in tumour-normal paired sequencing data, *Bioinformatics*, **28**, 167-175, doi: 10.1093/bioinformatics/btr629.

11. Duitama,J., Quintero,J.C., Cruz,D.F., Quintero,C., Hubmann,G., Foulquié-Moreno,M.R., Verstrepen,K.J., Thevelein,J.M., Tohme,J. (2014) An integrated framework for discovery and genotyping of genomic variants from high-throughput sequencing experiments, *Nucleic Acids Res*, **42**, 42, doi: 10.1093/nar/gkt1381.

12. Edmonson,M.N., Zhang,J., Yan,C., Finney,R.P., Meerzaman,D.M., Buetow,K.H. (2011) Bambino: a variant detector and alignment viewer for next-generation sequencing data in the SAM/BAM format, *Bioinformatics*, **27**, 865-866, doi:10.1093/bioinformatics/btr032.

13. Ewing,A.D., Houlahan,K.E., Hu,Y., Ellrott,K., Caloian,C., Yamaguchi,T.N., Bare,J.C., P'ng,C., Waggott,D., Sabelnykova,V.Y., ICGC-TCGA DREAM Somatic Mutation Calling Callenge participants, Kellen,M.R., Norman,T.C., Haussler,D., Friend,S.H., Stolovitzky,G., Margolin,A.A., Stuart,J.M., Boutros,P.C. (2015) Combining tumor genome simulation with crowdsourcing to benchmark somatic single-nucleotide-variant detection, *Nat Methods*, **12**, 623-630.

14. Fang,H., Narzisi,G., O'Rawe,J., Wu,Y., Rosenbaum,J., Ronemus,M., Iossifov,I., Schatz,M.C., Lyon,G.J. (2014) Reducing INDEL calling errors in whole-genome and exome sequencing data, *Genome Med*, **6**, 89, doi:10.1186/s13073-014-0089-z.

15. Flicek,P. *et al* (2014) Ensembl 2014, *Nucleic Acids Res*, **24**, doi: 10.1093/nar/gkt1196.

16. Gerstung,M., Papaemmanuil,E., Campbell,P.J. (2014) Subclonal variant calling with multiple samples and prior knowledge, *Bioinformatics*, **30**, 1198-1204, doi:10.1093/bioinformatics/btt750.

17. Goya,R., Sun,M.G., Morin,R.D., Leung,G., Ha,G., Wiegand,K.C., Senz,J., Crisan,A., Marra,M.A, Hirst,M., Huntsman,D., Murphy,K.P., Aparicio,S., Shah,S.P. (2010) SNVMix: predicting single nucleotide variants from next-generation sequencing of tumors, *Bioinformatics*, **26**, 730-6, doi:10.1093/bioinformatics/btq040.

18. Huang,W., Li,L., Myers,J.R., Marth,G.T. (2012) ART: a next-generation sequencing read simulator, *Bioinformatics*, **28**, doi:10.1093/bioinformatics/btr708.

19. Hansen,N.F., Gartner,J.J., Mei,L., Samuels,Y., Mullikin,J.C. (2013) Shimmer: detection of genetic alterations in tumors using next-generation sequence data, *Bioinformatics*, **29**, 1498-1503, doi:10.1093/bioinformatics/btt183.

20. Raczy,C., Petrovski,R., Saunders,C.t., Chorny,I., Kruglayak,S., Marguliers,E.H., Chuang,H.-Y., Källberg,M., Kumar,S.A., Liao,A., Little,K.M., Strömberg,M.P., Tanner,S.W. (2013) Isaac: Ultra-fast whole genome secondary analysis on Illumina sequencing platforms, *Bioinformatics*, **29**, 2041-3, doi:10.1093/bioinformatics/btt314.

21. Larson,D.E., Harris,C.C., Chen,K., Koboldt,D.C., Abbot,T.E., Dooling,D.J., Ley,T.J., Mardis,E.R., Wilson,R., Ding,L. (2013) SomaticSniper: identification of somatic point mutations in whole genome sequencing data, *Bioinformatics*, **18**, 311-317, doi:10.1093/bioinformatics/btr665.

22. Li,H., Durbin,R. (2009) Fast and accurate short read alignment with Burrows-Wheeler transform, *Bioinformatics*, **25**, 1754-1760.

23. Li,R., Li,Y., Kristiansen,K., Wang,J. (2008) SOAP: short oligonucleotide alignment program, *Bioinformatics*, **24**, 713-4, doi: 10.1093/bioinformatics/btn025.

24. Li,Y., Sidore,C., Kang,H.M., Boehnke,M., Abecasis,G.R. (2011) Low-coverage sequencing: Implications for design of complex trait association studies, *Genome Res*, **21**, 940-51.

25. Kim,S., Jeong,K., Bhutani,K., Ho Lee,J., Patel,A., Scott,E., Nam,H., Lee,H., Gleeson,J.G., Bafna,V. (2013) Virmid: accurate detection of somatic mutations with sample impurity inference, *Genome Biol*, **14**, R90, doi:10.1186/gb-2013-14-8-r90.

26. Qi,J., Zhao,F., Buboltz,A., Schuster,S.C. (2010) inGap: an integrated next-generation genome analysis pipeline, *Bioinformatics*, **26**, 127-129, doi:10.1093/bioinformatics/btp615.

27. R Core Team (2013) R: A Language and Environment for Statistical Computing. *R Foundation for Statistical Computing*. Vienna, Austria, http://www.R-project.org/.

28. Rashid,M., Robles-Espinoza,C.D., Rust,A.G., Adams,D.J. (2013) Cake: a bioinformatics pipeline for the integrated analysis of somatic variants in cancer genomes, *Bioinformatics*, **28**, 1811-1817, doi:10.1093/bioinformatics/bts271.

29. Roth,A., Ding,J., Morin,R., Crisan,A., Ha,G., Giuliany,R., Bashashati,A., Hirst,M., Turashvili,G., Olomui,A., Marra,M.A., Aparicio,S., Shah,S.P. (2012) JointSNVMix: a probabilistic model for accurate detection of somatic mutations in normal/tumour paired next-generation sequencing data, *Bioinformatics*, **28**, 907-913, doi:10.1093/bioinformatics/bts053.

30. Saunders,C.T., Wong,W.S.W., Swamy,S., Becq,J., Murray,L.J., Chetham,R.K. (2013) Strelka: accurate somatic small-variant calling from sequenced tumor–normal sample pairs, *Bioinformatics*, **28**, 1811-1817, doi:10.1093/bioinformatics/bts271.

31. Shiraishi,Y., Sato,Y., Chiba,K., Okuno,Y., Nagata,Y., Yoshida,K., Shiba,N., Hayashi,Y., Kume,H., Homma,Y., Sanada,M. Ogawa,S. Miyano,S. (2013) An empirical Bayesian framework for somatic mutation detection from cancer genome sequencing data, *Nucleic Acids Res*, **41**, e89, doi:10.1093/nar/gkt126.

32. SolSNP – Use of a modified Kolmogorov-Smirnov statistic and data filtering to call variants, available at: http://sourceforge.net/projects/solsnp/.

33. Stephens,P.J. *et al*. (2012) The landscape of cancer genes and mutational processes in breast cancer, *Nature*, **486**, 400-404, doi:10.1038/nature11017.

34. Torstenson,E.S., Li,B., Li,C. (2013) ASAP: an environment for automated preprocessing of sequencing data, *BMC Res Notes*, **6**, 5, doi:10.1186/1756-0500-6-5.

35. Vallania,F., Ramos,E., Cresci,S., Mitra,R.D., Druley,T.E. (2012) Detection of rare genomic variants from pooled sequencing using SPLINTER, *J Vis Exp*, **64**, 3943, doi:10.3791/3943.

36. Van der Borght,K., Thys,K., Wetzels,Y., Clement,L., Verbist,B., Reumers,J., van Vlijmen,H., Aerssens,J. (2015) QQ-SNV: single nucleotide variant detection at low frequency by comparing the quality quantiles, *BMC Bioinformatics*, **16**, 379, doi:10.1186/s12859-015-0812-9.

37. Yang,R., Nelson,A.C., Henzler,C., Thyagarajan,B., Silverstein,K.A.T. (2015) ScanIndel: a hybrid framework for indel detection via gapped alignment, split reads and de novo assembly, *Genome Med*, **7**, 1-12, doi:10.1186/s13073-015-0251-2.
